# The discovery of regional neurotoxicity-associated metabolic alterations induced by carbon quantum dots in brain of mice using a spatial metabolomics analysis

**DOI:** 10.1186/s12989-024-00580-y

**Published:** 2024-04-10

**Authors:** Min Chen, Siyuan Chen, Xinyu Wang, Zongjian Ye, Kehan Liu, Yijing Qian, Meng Tang, Tianshu Wu

**Affiliations:** Key Laboratory of Environmental Medicine and Engineering, Ministry of Education, School of Public Health, Southeast University, 210009 Nanjing, P.R. China

**Keywords:** AFADESI-MSI, Neuroinflammation, Redox disruption, Programmed cell death, Parkinson’s disease-like symptoms

## Abstract

**Background:**

Recently, carbon quantum dots (CQDs) have been widely used in various fields, especially in the diagnosis and therapy of neurological disorders, due to their excellent prospects. However, the associated inevitable exposure of CQDs to the environment and the public could have serious severe consequences limiting their safe application and sustainable development.

**Results:**

In this study, we found that intranasal treatment of 5 mg/kg BW (20 µL/nose of 0.5 mg/mL) CQDs affected the distribution of multiple metabolites and associated pathways in the brain of mice through the airflow-assisted desorption electrospray ionization mass spectrometry imaging (AFADESI-MSI) technique, which proved effective in discovery has proven to be significantly alerted and research into tissue-specific toxic biomarkers and molecular toxicity analysis. The neurotoxic biomarkers of CQDs identified by MSI analysis mainly contained aminos, lipids and lipid-like molecules which are involved in arginine and proline metabolism, biosynthesis of unsaturated fatty acids, and glutamine and glutamate metabolism, etc. as well as related metabolic enzymes. The levels or expressions of these metabolites and enzymes changed by CQDs in different brain regions would induce neuroinflammation, organelle damage, oxidative stress and multiple programmed cell deaths (PCDs), leading to neurodegeneration, such as Parkinson’s disease-like symptoms. This study enlightened risk assessments and interventions of QD-type or carbon-based nanoparticles on the nervous system based on toxic biomarkers regarding region-specific profiling of altered metabolic signatures.

**Conclusion:**

These findings provide information to advance knowledge of neurotoxic effects of CQDs and guide their further safety evaluation.

**Supplementary Information:**

The online version contains supplementary material available at 10.1186/s12989-024-00580-y.

## Background

Carbon-based nanomaterials have provided innovative solutions to some long-standing health problems in our modern society, as carbon and its allotropes are one of the most biologically important and abundant elements. CQDs are a kind of is one type of zero-dimensional carbon-based luminescent nanoparticles that possess obvious crystal lattices, which could be classified into carbon dots (CDs) [1]. A variety of advantages such as small size, biocompatibility, water solubility and physicochemical stability offer CQDs great opportunities for various applications in many fields, especially in biomedicine and neuroscience [2–4], which would allay public concerns if deployed on a large scale.

Apart from increasing application in the biomedical field, carbon nanoparticles, including carbon dots, have now been found to be derived from carbonaceous aerosol of atmospheric contaminants in the real environment [5] and human activities [6]. However, the accurately detecting environmental exposure of CDs or CQDs using current scientific detection technologies poses a challenge due to the intricate composition and dynamic transformation within complex environmental substrates (Jiang et al., 2022). Meanwhile, for biomedical applications or in real-world environment, the CQDs could enter living bodies and be transported to the central nervous system (CNS) by crossing the blood-brain barrier (BBB) or the olfactory nerve pathway [2, 7]. Recently, not only intranasal administration has been reported as a non-invasive approach to delivering drugs into the brain using nanomaterials, offering several advantages over conventional routes [8, 9], but also mimics the respiratory tract exposure of carbon-based nanoparticles obtained from airborne pollutants [10, 11]. Thus, the concerns regarding the safety and toxicity of CQDs must be addressed but the risk assessment of CQDs regarding the CNS is limited.

In recent years, a powerful imaging mass spectrometry tool is capable of directly overlaying the molecular information based on metabolomics analysis with a histopathological examination to correlatively monitor both distribution and toxicological mechanisms at the same time [12–14]. Among several tissue mass spectrometry imaging (MSI) techniques, the AFADESI-MSI is a method for broadly detecting metabolites with high sensitivity, wide dynamic range, and high specificity [15]. This study is the first one reporting a region-specifically altered metabolic molecules in the brain of mice exposed to CQDs using AFADESI-MSI. The molecular images of thousands of structure-specific and functionally relevant endogenous metabolites obtained from untargeted high-throughput AFADESI-MSI analysis not only helped us discover in situ neurotoxicity biomarkers but also provided insights into uncovering potential toxic mechanisms provide the molecular level [6]. Furthermore, some dysfunctional metabolic pathways in the pathological process of neurodegenerative disorders acquired from MSI-based metabolomics analysis [16] could provide insights into neurodegenerative disorders-associated metabolites and relevant metabolic enzymes under CQDs exposure. This study enhanced our comprehension of the brain regions and molecules that CQDs target by examining the incorporation of spatially resolved metabolites and relevant metabolic enzymes, which offers metabolically significant molecular initial events for further CQDs risk assessments and establishes the groundwork for constructing a nanomaterials adverse outcome pathways (AOP).

## Results

### Physiochemical characterization of water-soluble CQDs

**Fig. 1:**
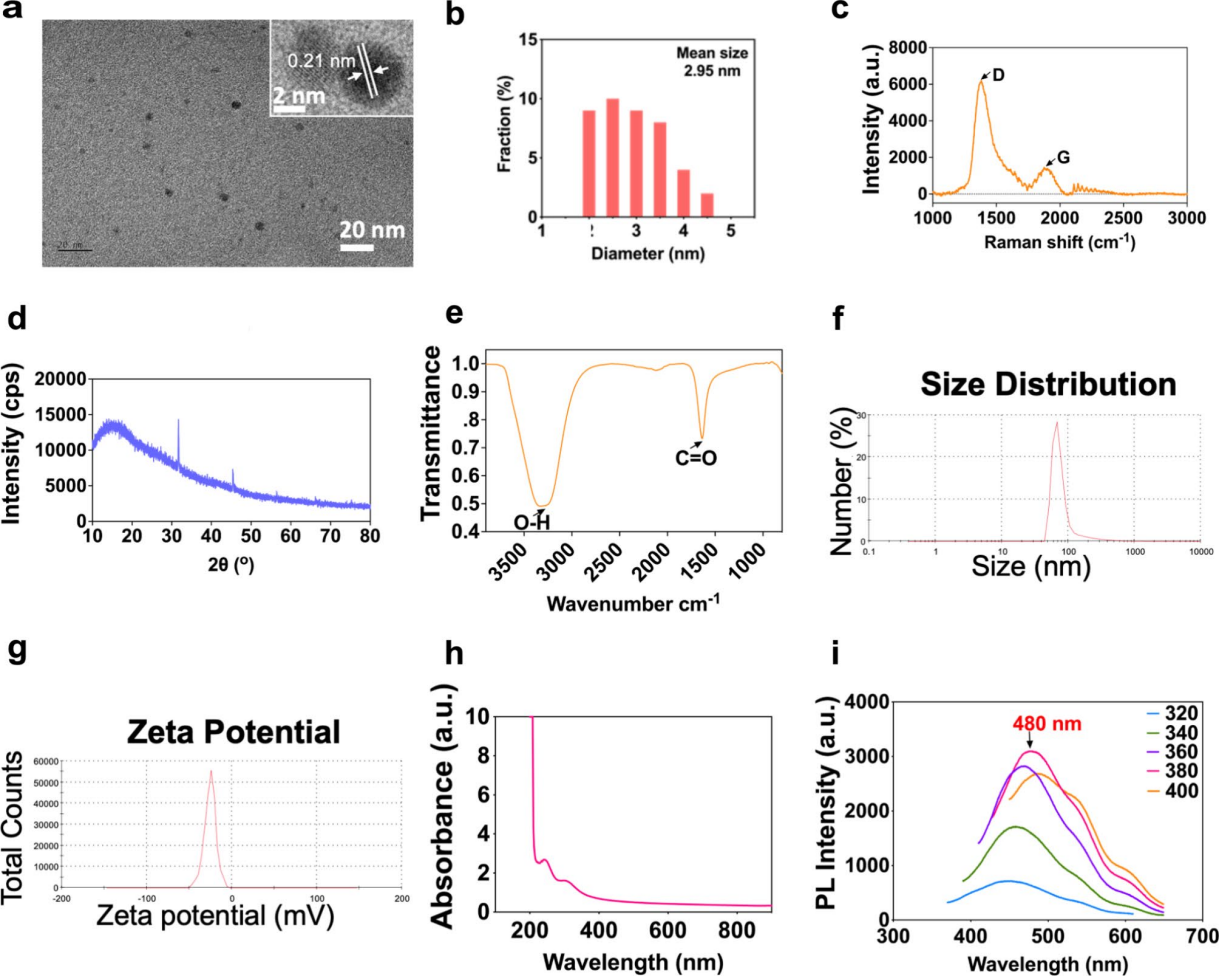
The physicochemical characterization of CQDs. (**a**) TEM image with HR-TEM image; (**b**) TEM size distribution; (**c**) Raman spectrum; (**d**) XRD spectrum; (**e**) FT-IR spectrum; (**f**) Hydrous particle diameters; (**g**) ξ-potentials; (**h**) UV-vis absorption spectrum; (**i**) Spectra of excitation wavelength increasing from 320 nm to 400 nm

The measurement of physicochemical properties of CQDs is of importance before conducting their risk assessment in living animals. High-resolution transmission electron microscope (HR-TEM) images suggested that CQDs were spherical particle and uniformed with average particle size of 2.95 nm (Fig. 1a). The average lattice spacing equaling 0.21 nm matched the in-plane lattice spacing of (100) facets graphite (Inserted photograph in Fig. 1a), which indicates the core of CQDs is graphitic [17, 18]. The Raman spectra of CQDs (Fig. 1b) showed two peaks at around 1375.1 cm^-1^ and 1884.2 cm^-1^ that are corresponded to D_disordered_ band and G_crystalline_ band, respectively. According to previous study, the D band originated from the vibrations of dangling carbon atoms in the termination plane of disordered graphite or glassy carbon, while the G band originated from the vibration of *sp*^*2*^-bonded carbon atoms in a two-dimensional hexagonal lattice. The large value of D to G intensity ratio (I_D_/I_G_) indicates the poor crystallization and low graphitization of CQDs because the I_D_/I_G_ as the ratio of *sp*^*3*^/*sp*^*2*^ carbon is characteristic of the extents of graphite [19]. The broad peak in the X-ray diffraction (XRD) spectrum (Fig. 1c) also attributed to the highly disordered carbon atoms, which is consistent with the graphite lattice spacing [20]. The fourier transform infrared (FT-IR) spectrum suggested stretching vibrations of O-H at around 3301 cm^-1^ and C = O at around 1630 cm^-1^ (Fig. 1d). The abundant hydrophilic functional groups on the surface of CQDs would ensure them to disperse well in any aqueous solutions. The hydrodynamic diameters of 0.5 mg/mL CQDs used in this study were measured to be approximately 84.2 nm, which was larger than TEM size (Fig. 1e). The ξ-potential of CQDs in the DI water was negative (Fig. 1f). More detailed physicochemical characteristics of CQDs were showed in Table S1.

### The intranasal treatment of CQDs caused mildly toxic effects in the brain

**Fig. 2:**
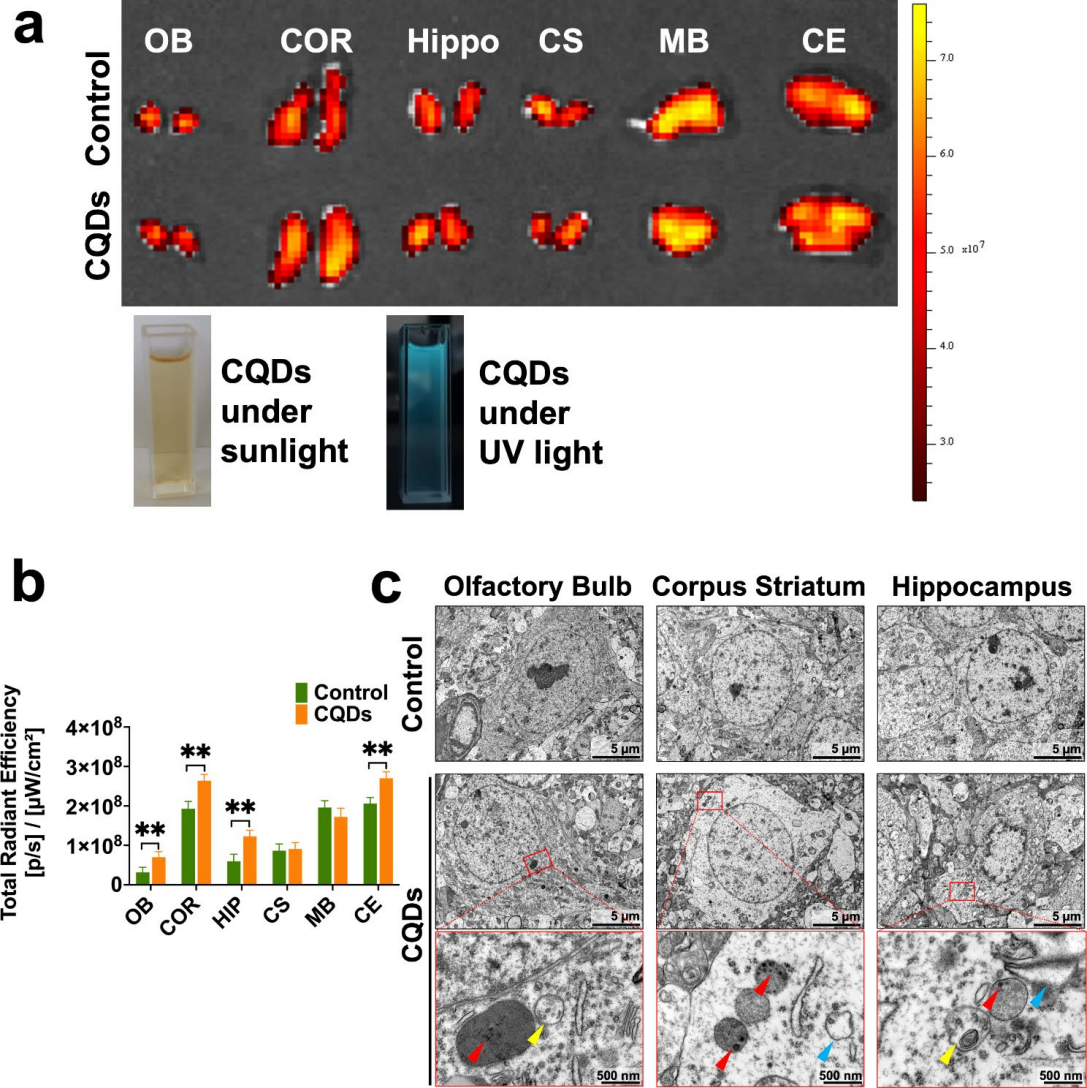
The uptake and biodistribution of CQDs in the brain of mice. (**a**) Representative fluorescence pictures of different regions of brain tissue in mice as well as pictures of CQDs under sunlight and UV light; (**b**) Quantitative results of radiant efficiency of CQDs; (**c**) Representative TEM images of OB, HIP and CS of the brain tissues showed the accumulation of CQDs in neuronal cells. The red arrows indicate accumulated CQDs in lysosome with membrane rupture, the yellow arrows indicate destroyed mitochondrion inside an autophagic vacuole (also known as autolysosome), the blue arrows indicate swollen endoplasmic reticulum. Olfactory bulb (OB), Cortex (COR), Hippocampus (HIP), Corpus Striatum (CS), Midbrain (MB) and Cerebellum (CE). Data are showed as mean ± SD of three independent experiments. (*P < 0.05, **P < 0.01, ***P < 0.001)

**Fig. 3:**
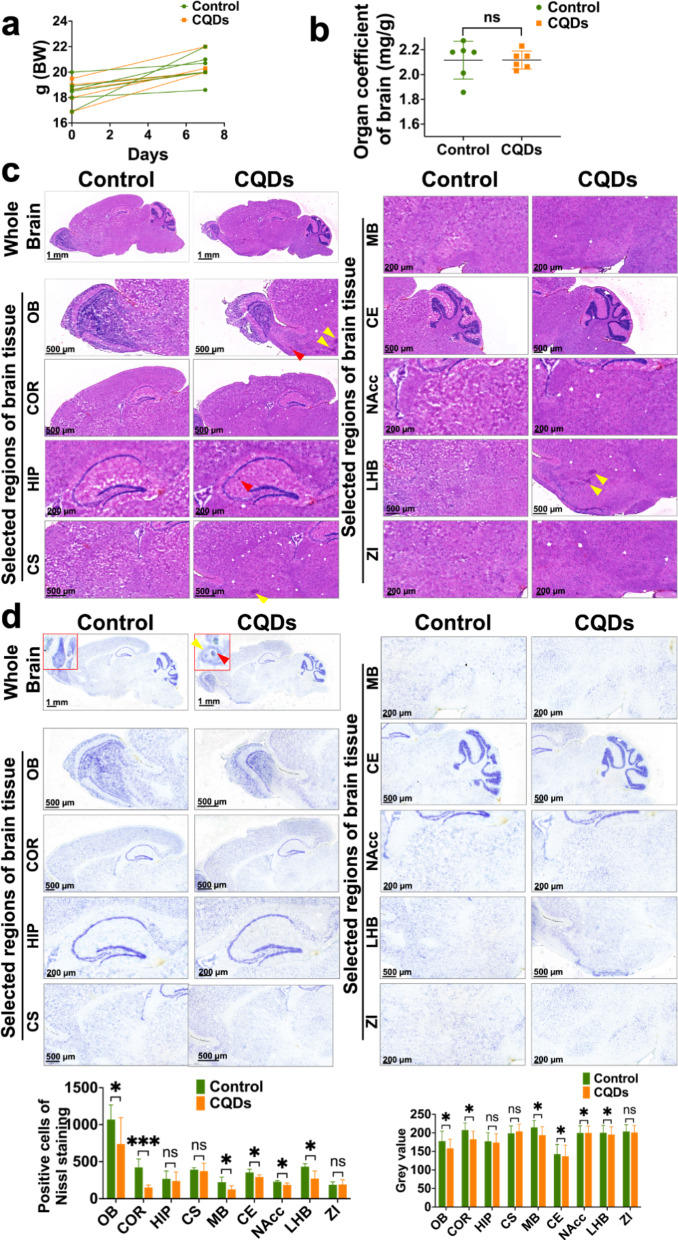
The general neurotoxic effects of CQDs in brain of mice. (**a**) The changes in the body weight of mice; (**b**) The changes in the organ coefficient of mice brain; (**c**) Representative histological images represented the whole brain tissue and different regions stained with H&E staining. The yellow arrows indicate bleeding spots, the red arrows indicate inflammatory cell infiltration; (**d**) Representative histological images represented the whole brain tissue and different regions stained with Nissl staining. The inserted images indicate magnified neurons with lighter Nissl bodies staining, vacuolar degeneration (red arrow) and blurry edges (yellow arrow) after CQDs exposure. Olfactory bulb (OB), Cortex (COR), Hippocampus (HIP), Corpus Striatum (CS), Midbrain (MB), Cerebellum (CE), Nucleus Accumbens (NAcc), Lateral Habenula Nucleus (LHB) and Zona Incerta (ZI)

The intranasal instillation of CQDs could stimulate their nose-to-brain delivery and respiratory tract exposure. Firstly, the exposure concentration of 5 mg/kg BW (20 µL/nose of 0.5 mg/mL) here was based on the dosage of CQDs applied in neuroscientific experiments being 2.4 µg/g BW in the mouse brain when the delivery efficiency of nose-to-brain translocation is approximately 2% and exposure for consecutive 7 days [21]. Meanwhile, it was also based on the internal exposure concentrations of carbon-based nanomaterials being µg/L/kg/m^3^ in water, solid media and air multiple with coefficient 1,000 [10, 11]. The exposed CQDs were measured in different regions of brain and mainly bioaccumulate in pericerebral parts (Fig. 2a and b). TEM images also suggested that CQDs accumulated in lysosomes and destroyed their normal membrane structure (Fig. 2c). During the experiment, no individual death as well as unchanged body weight and organ coefficient of brain were observed in all animals (Fig. 3a and b). However, the morphology of Olfactory bulb (OB) changed in CQDs-treated mice, and some bleeding spots or inflammatory cells infiltration were observed in OB, hippocampus (HIP), corpus striatum (CS) and lateral habenula nucleus (LHB) of brain exposed to CQDs (Fig. 3c). Additionally, the number of neurons stained with Nissl significantly decreased in some regions of brain following with diminished Nissl body, vacuolar degeneration, blurry edges and light staining (Fig. 3d).

### The region-specific molecule profiling and metabolic pathways in brain of mice treated with CQDs

An AFADESI-MSI strategy was used to high-throughput discover the neurotoxicity-associated metabolites altered by CQDs in the native brain state (Fig. S1). The quality of spatial metabolome data was firstly assured by a series of analysis (Fig. S2a–f), and then the global metabolites significantly changed by CQDs were explored belonging to super classes of lipids and lipid-like molecules, benzenoids, organic acids and derivatives, and organo-heterocyclic compounds (Fig. S2g–j), and enriching in metabolic pathways of arginine and proline metabolism, biosynthesis of unsaturated fatty acids, and D-glutamine and D-glutamate metabolism (Fig. S2k). The H&E staining brain tissue section in sagittal plane was divided into nine histological types based on the brain anatomy: OB, cortex (COR), HIP, CS, midbrain (MB), cerebellum (CE), nucleus accumbens (NAcc), LHB and zona incerta (ZI) to discover region-specific metabolites altered by CQDs in the brain of mice (Fig. S3a–c). The most affected metabolic pathways by CQDs were arginine and proline metabolism in brain regions of OB, COR, CS, CE and NAcc, while biosynthesis of unsaturated fatty acids in brain regions of HIP, MB, LHB and ZI (Fig. S3d). Additionally, one interesting discovery is the enriched metabolic pathways were different among different brain regions both in the control and in mice treated with CQDs, when we merely compared the discriminating metabolites in brain regions of OB, HIP, CS and MB (Fig. S4).

### The validation on discovery of region-specific metabolites and relevant metabolic enzymes significantly changed by CQDs

**Fig. 4:**
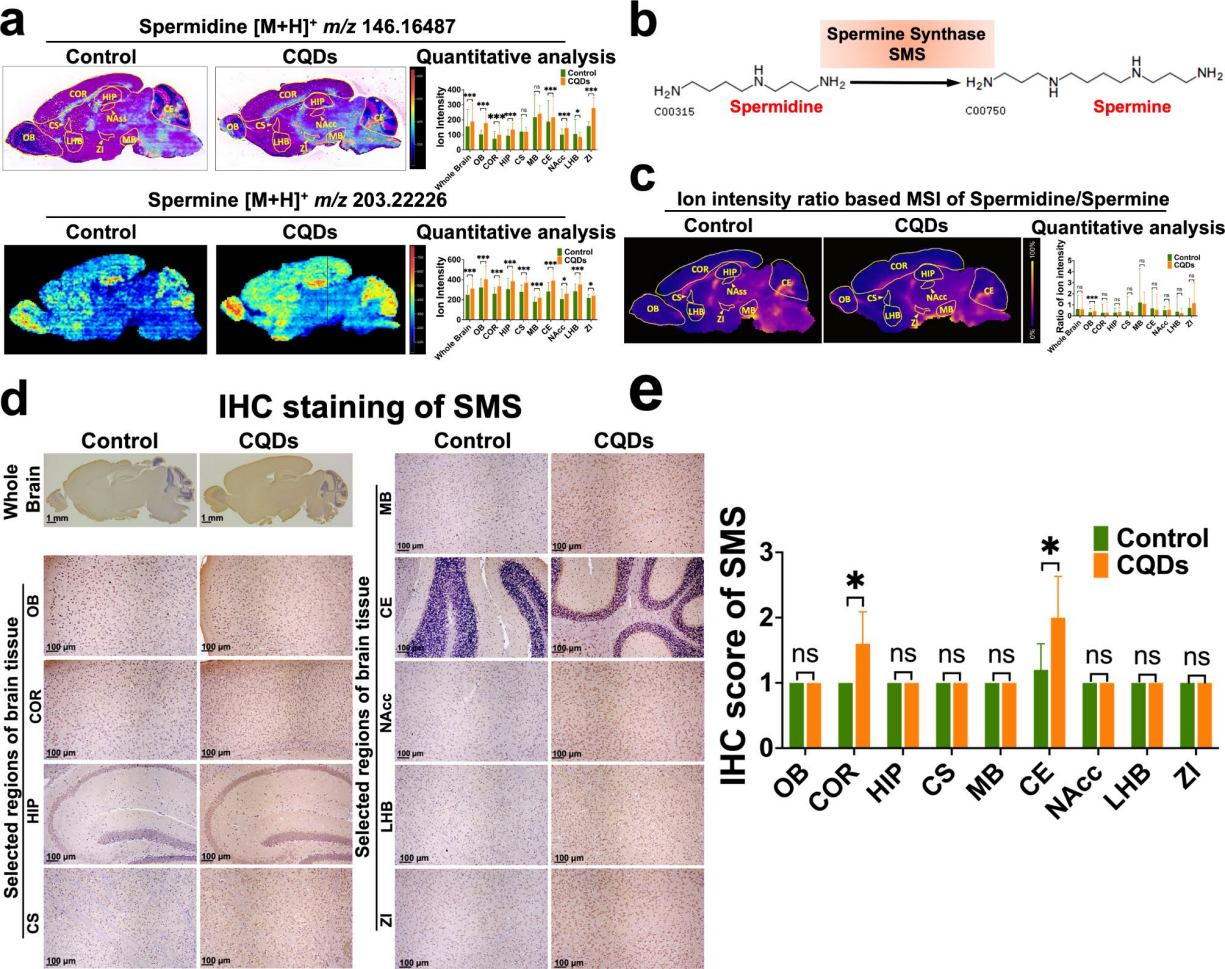
In situ visualization of critical metabolites and metabolic enzymes in arginine and proline metabolism changed by CQDs in brain of mice. (**a**) MSI and H&E overlay images or MS images and levels of spermidine and spermine in different regions of brain; (**b**) SMS-mediated metabolic process of converting spermidine to spermine; (**c**) The newly constructed MSI based on the ion intensity ratio of converting spermidine to spermine; (**d**, **e**) Representative IHC staining showed the expression of SMS in different regions of brain tissue section and its IHC score. Olfactory bulb (OB), Cortex (COR), Hippocampus (HIP), Corpus Striatum (CS), Midbrain (MB), Cerebellum (CE), Nucleus Accumbens (NAcc), Lateral Habenula Nucleus (LHB) and Zona Incerta (ZI). Data are showed as mean ± SD of three independent experiments. (*P < 0.05, **P < 0.01, ***P < 0.001)

Spermidine and spermine are important polyamines in arginine and proline metabolism, and have prominent neuroprotective effects and anti-inflammatory effects through preserving mitochondrial function, preventing DNA damages in rodent models [22]. The MSI and H&E overlay images not only facilitated to extract region-specific metabolite profiles, but also elicited that the ion intensities of spermidine in brain regions other than CS and MB in CQDs group were significantly higher than the control, while spermine significantly up-regulated in all selected brain regions after CQDs exposure (Fig. 4a). Since the spermine synthase (SMS) is essential for spermine being synthesized from the reaction of spermidine with decarboxylated S-adenosylmethionine (Fig. 4b), its alternation could be reflected by the pixel-by-pixel ion intensity ratio of MS images of spermidine and spermine, and LHB and ZI regions of brain exposed to CQDs possessed a significantly different ion-intensity ratio than the control (Fig. 4c). Furthermore, the Immunohistochemistry (IHC) staining of SMS indicated that the up-regulated SMS was expressed in almost all selected regions of brain of mice exposed to CQDs, which was accord to the spatial distribution of spermidine/spermine (Fig. 4d and e). Meanwhile, the levels of many other metabolites in arginine and proline metabolism in different regions of brains were significantly changed by CQDs as well (Fig. S5a).

**Fig. 5:**
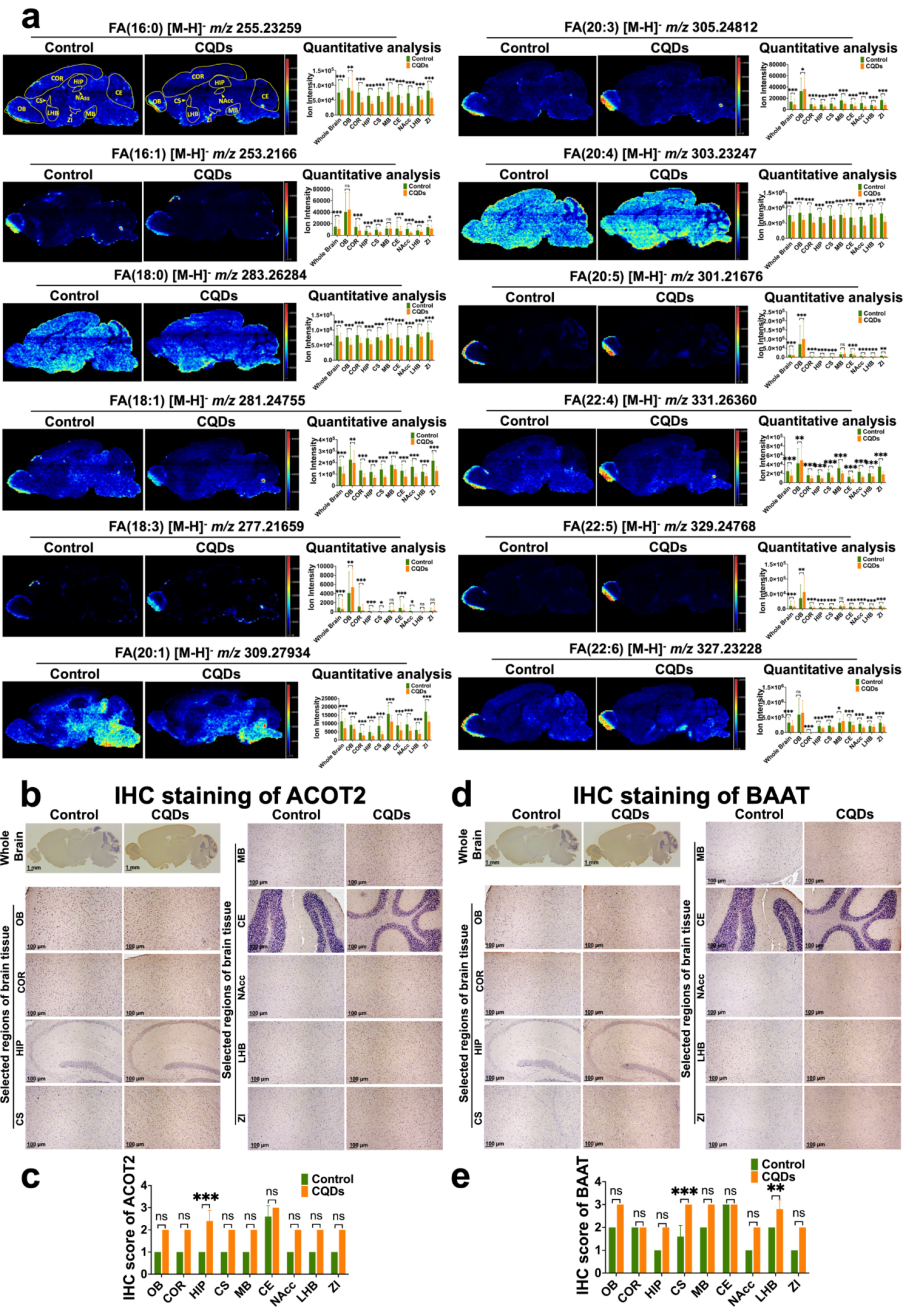
In situ visualization of critical metabolites and metabolic enzymes in biosynthesis of unsaturated fatty acids changed by CQDs in brain of mice. (**a**) MS images and levels of fatty acids in different regions of brain tissue section; Representative IHC staining showed the expression of ACOT2 (**b**, **c**) and BAAT (**d**, **e**) in different regions of brain tissue section and their IHC scores. Olfactory bulb (OB), Cortex (COR), Hippocampus (HIP), Corpus Striatum (CS), Midbrain (MB), Cerebellum (CE), Nucleus Accumbens (NAcc), Lateral Habenula Nucleus (LHB) and Zona Incerta (ZI). Data are showed as mean ± SD of three independent experiments. (*P < 0.05, **P < 0.01, ***P < 0.001)

The unsaturated fatty acids have been recognized as essential components for the CNS, where they play multiple roles in the health and diseased brain [23]. MS images demonstrated that the ion intensities of representative fatty acids (Table S2) were weaker in selected brain regions of mice treated with CQDs than the control (Fig. 5a). In the pathway of biosynthesis of unsaturated fatty acids, ACOT2 and BAAT are two key metabolic enzymes for the biosynthesis of important unsaturated fatty acids, including arachidonic acid, docosahexaenoic acid (DHA), palmitic acid and stearic acid [24]. Notably, the spatial expression of acyl-CoA thioesterase 2 (ACOT2) and bile acid-Coenzyme A: amino acid N-acyltransferase (BAAT) were consistent with the distribution of down-regulated fatty acids in brain of mice treated with CQDs (Fig. 5b–e).

**Fig. 6:**
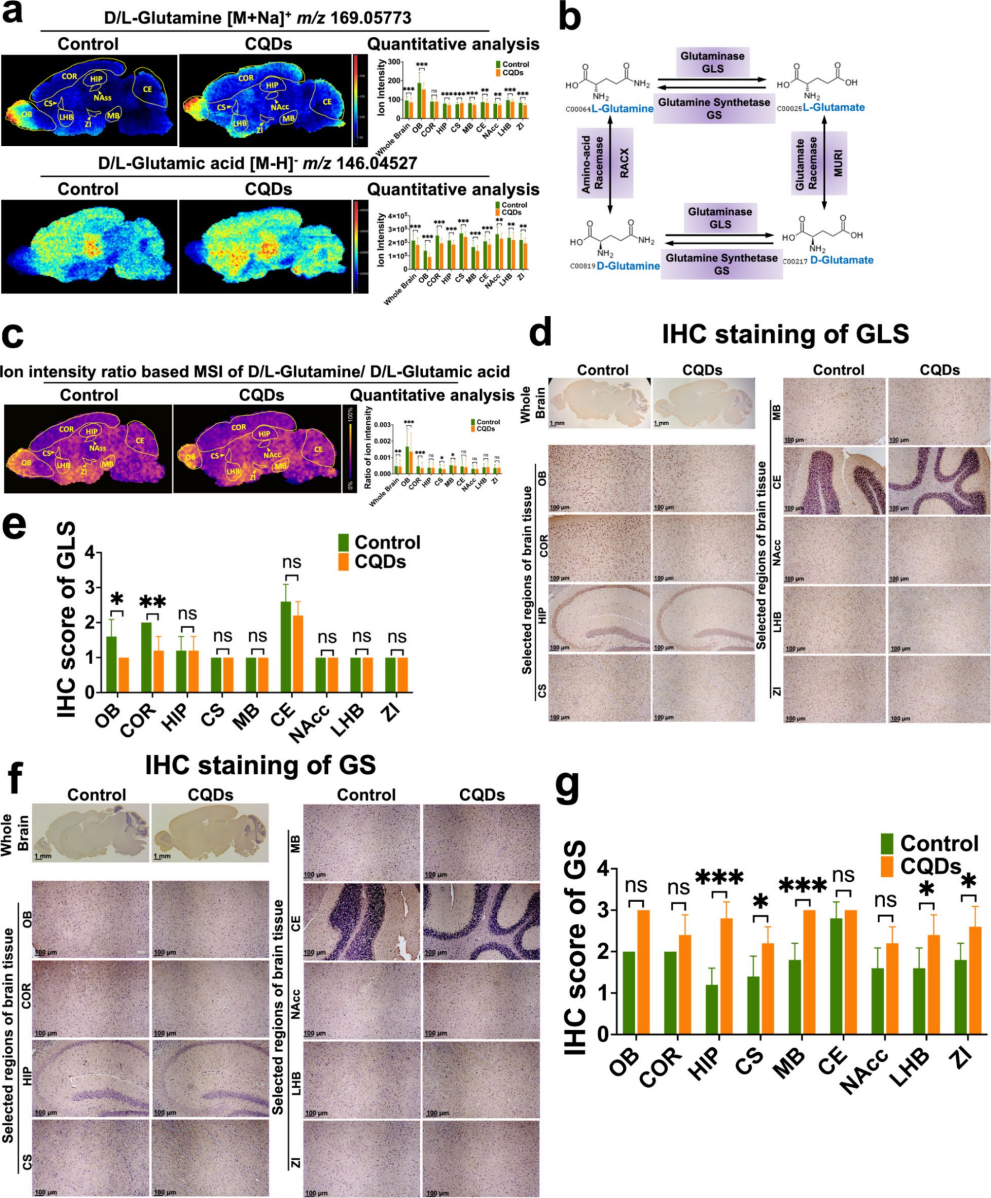
In situ visualization of critical metabolites and metabolic enzymes in glutamine and glutamate metabolism changed by CQDs in brain of mice. (**a**) MS images and levels of D/L-glutamine and D/L-glutamate in different regions of brain; (**b**) GLS/GS-mediated metabolic process of interconversion between D/L-glutamine and D/L-glutamate; (**c**) The newly constructed MSI based on the ion intensity ratio of D/L-glutamine and D/L-glutamate; Representative IHC staining showed the expression of GLS (**d**, **e**) and GS (**f**, **g**) in different regions of brain tissue section and their IHC scores. Olfactory bulb (OB), Cortex (COR), Hippocampus (HIP), Corpus Striatum (CS), Midbrain (MB), Cerebellum (CE), Nucleus Accumbens (NAcc), Lateral Habenula Nucleus (LHB) and Zona Incerta (ZI). Data are showed as mean ± SD of three independent experiments. (*P < 0.05, **P < 0.01, ***P < 0.001)

As small molecule amino acids, glutamine and glutamate are indispensable components of many critical biological processes, including protein synthesis, regulation of brain function, and maintenance and repairment of tissues [25]. MS images indicated that glutamine and glutamate both down-regulated in selected brain regions of mice exposed to CQDs, apart from the level of glutamine being elevated in CS region after CQDs exposure (Fig. 6a). In the glutamine and glutamate metabolism pathway, the glutamine catabolism is mediated by glutaminase (GLS) through hydrolyzing glutamine to glutamate, while glutamate is also the hydrolysis product of glutamine regulated by glutamine synthetase (GS) (Fig. 6b). The in situ intensity ratio of glutamine to glutamate were found to be significantly increased after CQDs exposure (Fig. 6c), which indicated enhanced glutamine hydrolysis rate or reduced glutamine synthesis rate. The subsequent IHC assay suggested down-regulated GLS and up-regulated GS in brain tissues of mice treated with CQDs compared with the control, in good consistence with the distribution of glutamine and glutamate (Fig. 6d–g). Overall, the IHC validation in the brain tissue section adjacent to that used in MSI indicated five enzymes of SMS, ACOT2, BAAT, GLS and GS were influenced greatly by altered metabolic features in CQDs-treated brain (Table S3).

### The alterations on metabolites induced by CQDs resulted in neuronal damages, especially programmed cell deaths (PCDs) at a region-specific manner in the brain of mice

**Fig. 7:**
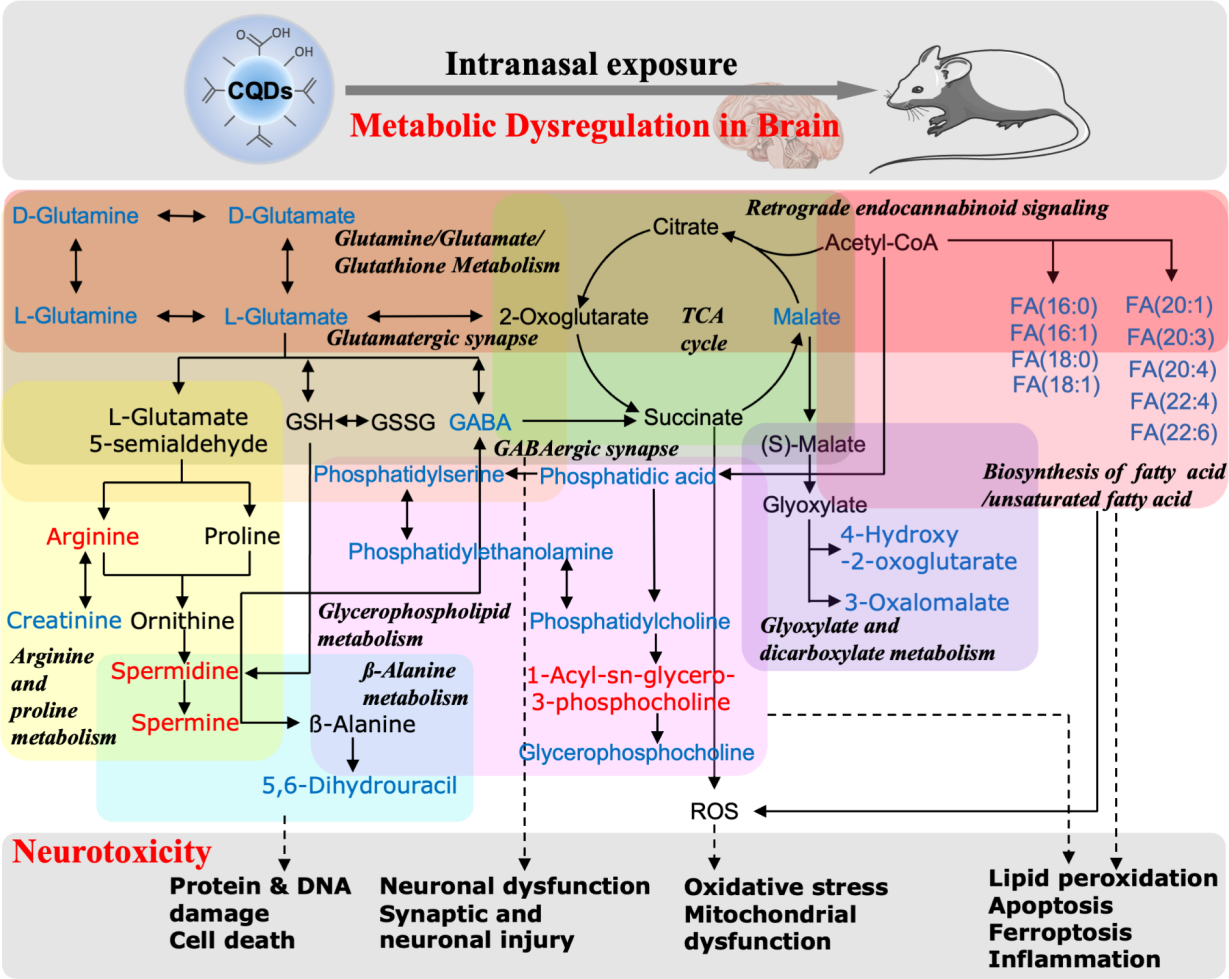
Schematic diagram on the overview of identified metabolic pathways dysregulated by CQDs in brain of mice

Apart from three pathways mentioned above that were altered by CQDs, levels of metabolites in metabolic pathways of ß-alanine metabolism mainly in OB region (Fig. S5b), Trichloroacetic acid (TCA) cycle mainly in HIP region (Fig. S5c), neuroactive ligand-receptor interaction (Fig. S5d) and glycerophospholipid metabolism and GhRN signaling pathway mainly in CS and MB regions (Fig. S5e) were discovered to significantly affected by CQDs in the brain tissues, and ultimately induced several neuronal damages (Fig. 7). Since most metabolites altered by CQDs are lipids and lipid-like molecules (Fig. S2j and S6a), MS images indicated that critical lipids in classes of fatty acyls (Fig. S6b), glycerolipids (Fig. S6c), prenol lipids (Fig. S6d), sphingolipids (Fig. S6e) and steroids and steroid derivative (Fig. S6f) were significantly changed by CQDs in selected brain regions. Lipids are paramount important to cellular homeostasis and the interference of lipid homeostasis under CQDs exposure was related with several neurotoxic effects, including inflammation responses, including enhancements on pro-inflammatory cytokines interleukin-1ß (IL-1ß) and tumor necrosis factor-α (TNF-α), and decreased anti-proinflammatory cytokine interleukin-10 (IL-10) (Fig. S7a–c), oxidative stress damages indicated by increased MDA levels, reduced glutathione (GSH) depletion and increased NADP+/NADPH ratio (Fig. S7d–f), DNA and mitochondrial damages (Fig. 2c), which could result in PCDs [26, 27].

**Fig. 8:**
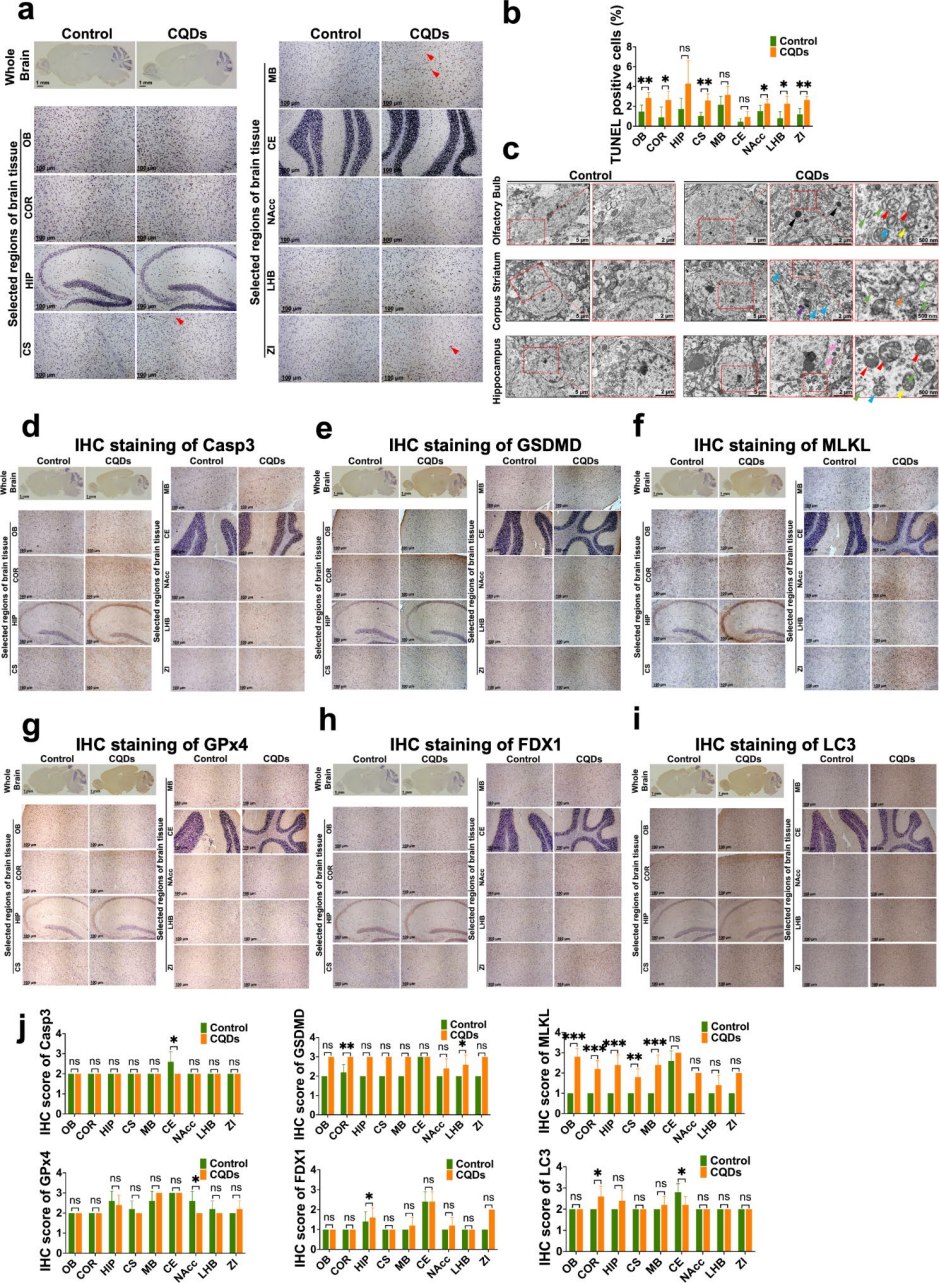
CQDs induced multiple programmed cell deaths in brain of mice. (**a**, **b**) Representative images and the quantitative results of TUNEL positive cells indicating apoptotic and dead cells in different regions of brain; (**c**) Representative TEM images of OB, HIP and CS of the brain tissues showed remarkable alternations in neuronal cells after CQDs exposure. The black arrows indicate accumulated CQDs in lysosome with broken membrane, the red arrows indicate reduction and disappearance of mitochondrial ridge, the yellow arrows indicate collapse of mitochondrial membrane, the blue arrows indicate destroyed mitochondrion inside an autophagic vacuole (also known as autolysosome), the green arrows indicate dilated endoplasmic reticulum, the purple arrows indicate apoptotic body, the orange arrows indicate swollen mitochondria with broken ridge, the pink arrows indicate shrunken and dense mitochondria; Representative IHC staining showed the expression of caspase 3 (**d**), GSDMD (**e**), MLKL (**f**), GPx4 (**g**), FDX1 (**h**) and LC3 (**i**) in different regions of brain tissue section, as well as their IHC scores (**j**). Olfactory bulb (OB), Cortex (COR), Hippocampus (HIP), Corpus Striatum (CS), Midbrain (MB), Cerebellum (CE), Nucleus Accumbens (NAcc), Lateral Habenula Nucleus (LHB) and Zona Incerta (ZI). Data are showed as mean ± SD of three independent experiments. (*P < 0.05, **P < 0.01, ***P < 0.001)

Cell death triggered by CQDs was confirmed by not only terminal-deoxynucleotidyl transferase mediated nick end labelling (TUNEL) staining (Fig. 8a and b), but also TEM images showing typical morphological changes relevant to several PCDs (Figs. 2c and 8c). Specially, the hippocampal cell presented characteristics of apoptosis, including cell shrinkage, karyokinesis, chromatin granules concentrating to heterochromatin areas and forming chromatin blocks (Fig. 2c). The neuronal cells in CS also presented characteristics of necroptosis, like organelles swelling, and pyroptosis, such as chromatin pyknosis, cell membrane vesiculation, endoplasmic reticulum expansion, and DNA fragmentation, while neuronal cells in HIP reflect alterations of ferroptosis, including decreased size of mitochondria, increased density of bilayer membrane, break membrane and disappeared ridge, and autophagy with formation of autolysosomes involved in damaged mitochondrion (Fig. 8c). Moreover, typical biomarkers, including caspase 3 and caspase 8 for apoptosis (Fig. 8d, S8a and S8b), gasdermin D (GSDMD) and caspase 1 for necroptosis (Fig. 8e, S8c and S8d), mixed lineage kinase domain-like (MLKL) and receptor interacting protein-1 (RIP1) for pyroptosis (Fig. 8f, S8e and S8f), glutathione peroxidase 4 (GPx4) and acyl coenzyme A synthetase long chain family member 4 (ACSL4) for ferroptosis (Fig. 8g, S8g and S8h), ferredoxin 1 (FDX1) and heat shock protein 70 (HSP70) for cuproptosis (Fig. 8h, S8i and S8j) and microtubule associated protein 1 A/1B light chain 3 (LC3) and beclin-1 for autophagy (Fig. 8i, S8k and S8l) were all altered to some extents in brain of mice treated with CQDs (Fig. 8j).

**Fig. 9:**
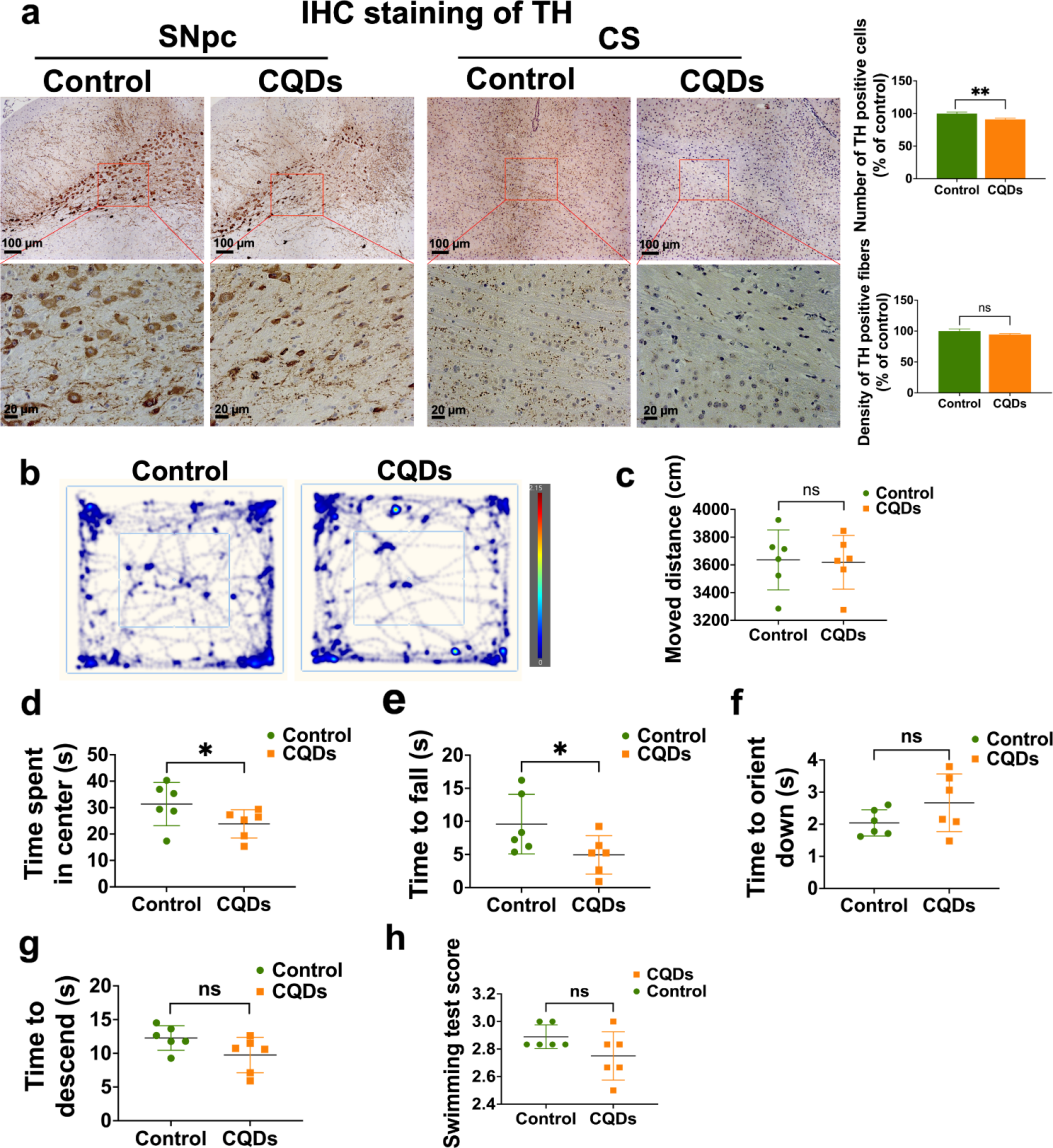
CQDs caused PD-like degeneration in mice. (**a**) Representative IHC staining images and quantitative results of TH positive cells in SNpc and TH positive terminals in striata; (**b**) Representative track and heatmap images in the open-field test, and the quantitative results of parameter analysis including total moved distance (**c**) and time spent in center (**d**); (**e**) The time to fall in suspension test; (**f**, **g**) The time to orient down and time to descend in pole-climbing test; (**h**) The scores of swim test. Data are showed as mean ± SD of three independent experiments. (*P < 0.05, **P < 0.01, ***P < 0.001)

Since PCDs are hallmarks of numerous brain disorders, especially neurodegenerative diseases that are always correlated with unwanted loss of neuronal cells [28]. We took Parkinson’s disease (PD) as an example since PCDs triggered by CQDs mostly occurred in COR, HIP, CS and MB regions of brain. The smaller number of tyrosine hydroxylase (TH) positive cells in the substantia nigra pars compacta (SNpc) of brain of mice exposure to CQDs compared to the control but the density of TH positive fibers in the striata did not decrease after CQDs exposure (Fig. 9a). Moreover, several PD relevant neurobehaviors, including autonomic movement (Fig. 9b–d), suspension time (Fig. 9e), pole-climbing (Fig. 9f and g) and swim scores (Fig. 9h) were altered by CQDs to some degrees.

## Discussion

The carbon nanoparticles, mainly less than 10 nm, are composed of CQDs and are formed through various pathways, some of which can be naturally released into the environment and others of which are manufactured [29]. In this study, exposure administration of CQDs simulated both airborne nanoparticles, nose-to-brain drug delivery [5, 9, 21], and treatment time is consistent with acute exposure to environmental pollutants or short-term drug use in order to assess the risk of increasing exposure of CQDs to the environment and the public and to improve their biosafe application and sustainable development. Intranasal exposure of CQDs accumulated throughout brain tissue, especially in the outer shell of the brain, which may be due to nerve conduction to the CNS following intranasally exposed nanoparticles [7]. The uneven biodistribution of CQDs in the brain could lead to region-specific neurotoxic effects due to different levels of vascularization in different areas of the brain [30]. Here, a novel technique called the AFADESI-MSI approach was used to provide data on region-specific metabolites that could predict differentially expressed metabolic enzymes in CQDs-exposed brains without previously predicting specific molecules of interest, which contributes to expand our understanding of the complex mechanisms of CQDs causing neurotoxic effects at the metabolite level.

The AFADESI-MSI data revealed region-specific metabolites and relevant metabolic pathways affected by CQDs, mainly arginine and proline metabolism, biosynthesis of unsaturated fatty acids, and D-glutamine and D-glutamate metabolism. The findings differed from the data obtained from oral exposure to a single dose of CQDs of 2 µg/g BW and mainly showed an altered pathway of energy production from aerobic to glycolytic metabolism according to brain distribution in male BALB/c mice [31]. It has now been found that some metabolic enzymes altered by CQDs, i.e. SMS, ACOT2, BAAT, GLS and GS, are recognized as important nodes in metabolic pathways regulating toxic metabolic reactions, which could be considered as potential toxic biomarkers of CQDs. When the expression of these enzymes was altered by xenobiotics like nanoparticles, several health problems occurred, including brain disorders due to SMS deficiency [32], apoptosis and inflammation due to dysregulated ACOT2 and BAAT [33, 34], neurological diseases and neuroinflammation induced by overexpression of GLS and GS [35, 36]. Targeted inhibition or induction of these enzymes or relevant metabolites may shed light on the preventive intervention of CQDs due to their neurotoxicity.

Since altered metabolic enzymes as well as lipids and lipid-like molecules that are mainly discriminating metabolites under the CQDs exposure are all closely related to neuroinflammation, the inflammatoryresponses to CQDs and inflammatory-relevant PCDs in the brain were primarily evaluated. When the neuroinflammatory reaction to CQDs in brain of mice was evidenced by the inflammatory cell infiltration, enhanced levels of pro-inflammatory cytokines IL-1ß and TNF-α while decreased level of anti-inflammatory cytokine IL-10. Meanwhile, inflammatory-relevant PCDs, including pyroptosis, apoptosis, necroptosis, ferroptosis, cuproptosis and autophagy were all discovered. Recently, the extensive crosstalk among pathways of apoptosis, necroptosis and pyroptosis were evidenced to established a new concept of PANoptosis in neuronal cell death that could be triggered by PANoptosome [37]. The PANoptosome protein complex specifically encompasses key signaling molecules, including caspase 1, GSDMD, gasdermin E (GSDME) for pyroptosis, caspase 8, caspase 3, FADD for apoptosis, and RIPK1, MLKL for necroptosis [38], which were all found to be altered by CQDs in this study.

The induction of PCDs might be related with organelles of mitochondria and lysosome damaged by CQDs in the brain. The mitochondrial impairments are sensitive to several carbon-based nanomaterials, resulting in the occurrence of PCDs [39, 40], while the lysosomal damage disrupts intracellular clearance to leak cell death triggered contents [41]. As autolysosomes were obviously observed in several neuronal cells in OB, HIP and CS brain regions of mice treated with CQDs, lysosomal overload could result from the excessive deposition of CQDs, which have been reported in several nanotoxicological studies [42]. Additionally, the alternations of metabolites levels could directly lead to cell death. Recently, researchers found a cell death as disulfidptosis that are regulated by disulfide stress under the excessive accumulation of cystine [43]. Our findings suggested the increase in level of L-cysteine caused by CQDs, probably resulting in novel PCDs that needs to be assessed in further studies.

The consequences of PCDs might be associated with the pathogenesis of neurodegenerative diseases, such as amyotrophic lateral sclerosis (ALS), Alzheimer’s disease (AD), PD and Huntington’s disease (HD), leading to unwanted loss of neuronal cells and function [28]. In this study, we further assessed the potential of PD-like neurodegeneration caused by CQDs according to observed mitochondria damages, oxidative stress, multiple PCDs and neuroinflammation which are all common features in PD [44]. The findings suggested that CQDs exposure caused slight dopaminergic neuron loss and neurobehavioural behaviors in mice, but severe PD-like symptoms, such as resting tremor, muscle rigidity, postural gait disturbances were not found, which indicated the PD-like neurodegenerative effects of CQDs are mild and at the early stage. However, it is still of worth to be attention because the aberrant activation of CQDs-mediated toxicity pathways, including oxidative stress, PCDs and neuroinflammation might result in progressive brain damages and cognitive impairments.

In this study, the AFADESI-MSI data suggest that arginine and proline metabolism, glutamine and glutamate metabolism and biosynthesis of unsaturated fatty acid are major metabolic pathways influenced by CQDs and then to different types of PCDs could lead to neurodegenerative disorders associated with PD. Although the specific toxicity mechanisms of CQDs causing brain damage are limited, the identified biomarkers of metabolites and relevant metabolic enzymes are of value for further risk assessment of CQDs in the CNS. Further research is needed to investigate the aberrant metabolic pathways associated with neurotoxicity caused by CQDs. Since the particle size is one of important physicochemical characteristics of QDs to manipulate their toxicity [45], further studies would consider to unveil whether and how the dimensions of CQDs changing their toxicity profile. Current understanding of these pathways is limited as screening has only identified potential metabolic vulnerabilities and a small number of dysregulated metabolic enzymes. However, given the large number of potential enzymes involved in metabolic pathways, a more comprehensive analysis is required. Furthermore, future mechanistic studies on PCDs should be conducted at the single-cell level. This approach would allow a more accurate assessment of the observed phenotypes of multiple PCDs, as it would account for the possibility that different cellular subclusters undergo distinct PCDs in response to CQDs.

## Conclusion

Assessing the risk of CQDs within the carbonaceous aerosol of highly concentrated urban particulate pollutants and in the applications of neuroscience and medical neurobiology is of significant importance. A novel technique, AFADESI-MSI, was employed to draw a comprehensive depiction of the toxic responses in the brains of mice following intranasal exposure to CQDs. This technique enables the identification of metabolites and relevant metabolic pathways, thereby providing substantial evidence to investigate the potential mechanisms underlying the neurotoxicity induced by CQDs in the CNS. According to the atlas, exposure to CQDs could induce mitochondrial and lysosomal damages, inflammatory responses and oxidative stress, as well as multiple PCDs, and then likely lead to PD-like degeneration, mainly through disruption of amino and lipid metabolisms in different regions of brain tissue. This study not only reminded researchers to pay more attention to mechanism-based risk assessment in CQDs, but also provided important insights into the discovery of metabolic enzymes related to neurodegenerative diseases under CQDs treatment in real-world and biomedical settings.

## Materials and methods

### Reagents

The MLKL, FDX1, GSDMD, p-MLKL, RIP1, p-RIP1, GS, GLS, ACOT2, BAAT, GPx4, ACSL4, LC3, TH and GAPDH primary antibodies were purchased from ABclonal Technology (Wuhan, China). The SMS, HSP70, caspase3 and capase8 primary antibody were purchased from ZENBIO Technology (Chen du, China). The capase1 primary antibody was purchased from ThermoScientific Technology (Massachusetts, United States). The beclin-1 primary antibody was purchased from Cell Signaling Technology (Boston, United States). The details of antibodies were shown in Table S4. IL-1ß, TNF-α and IL-10 commercial reagent kits were purchased from Yi Fei Xue Biotechnology (Nanjing, China). Lipid Peroxidation MDA Assay Kit, Reduced glutathione/Oxidized glutathione (GSH/GSSG) Assay Kit and NADP+/NADPH Assay Kit with WST-8 were purchased from Beyo-time Biotechnology (Shanghai, China).

### The physicochemical characterizations of CQDs

In this study, the CQDs was purchased from XFNANO Materials Tech Co., Ltd. (Nanjing, China) (Product No. XF253, http://www.xfnano.com). We firstly evaluate the physicochemical characterizations of CQDs. An electron microscope (JEM-2100, JEOL Ltd. Japan) was used to take HR-TEM images. The structure of CQDs was measured by XRD (D8 Advance Diffractometer). The FT-IR of CQDs was analyzed using Nicolet iS10 spectrometer (Thermo, USA). The Raman of CQDs was analyzed using Labram HR800 (Horiba Jobin-Yvon, Paris, France). A Malcern Zetasizer Nano ZS instrument (Zetasizer Nano-ZS90, Malvern, UK) was used to measure the dynamic light scattering (DLS) and surface ξ-potential of CQDs.

### Animals and treatment

Total 12 (6 mice per group) male 8 weeks C57BL/6 mice were purchased from Zhejiang Academy of Medical Sciences (Zhejiang, China). All mice were housed in stainless steel cages in a ventilated animal facility at a temperature maintained in 22 ± 2 ^o^C and relative humidity of 65 ± 10% under a 12 h light/dark cycle, and feed with sterilized food and distilled water. All mice were treated humanely throughout the experimental period, and procedures were approved by the Animal Experimental Ethics Committee of Southeast University (Nanjing, China) in strict accordance with the Guidelines for Care and Use of Laboratory Animals of Southeast University.

Experiments were According to the bio-application of CQDs in laboratories [18, 46]. With the help of an ultrasonic instrument, CQDs were dissolved in normal saline, and sonicated for 30 min every day before exposure to ensure that CQDs were evenly dispersed in normal saline. When the mice were awake, the solution was dropped into the nasal cavity of the mice, and the mice ingested CQDs through normal breathing. The experimental group was intranasally instilled with 5 mg/kg BW CQDs for 7 consecutive days, while the control group was given normal saline.

At the end of exposure, mice were sacrificed by inhaling carbon dioxide and then quickly removed and weighted their brains. One brain tissue sample of each group was transferred to specific embedding glue and stored at -80 ^o^C for AFADESI-MSI analysis in Shanghai Luming Biological Technology Co. Ltd. (Shanghai, China). OB, HIP and CS were dissected and fixed in 2.5% glutaraldehyde to do TEM examination. Meantime, OB, COR, HIP, CS, MB and CE were dissected and stored in liquid nitrogen for following experiments. The rest of brain tissues were fixed in 4% (w/v) paraformaldehyde for following experiments.

### Histology examinations

Brain sections were stained with H&E and toluidine blue. The TUNEL analysis was used to detect the apoptotic and dead cells in brain. A light microscope was used to take images in five non-overlapping randomly selected fields of OB, HIP, CS, COR, MB, CE, NAcc, LHB and ZI of each brain tissue section, and then count the number of TUNEL positive cells.

### Immunohistochemistry

Brain sections were incubated with SMS, GLS, GS, ACOT2, BAAT, caspase 3, GSDMD, MLKL, GPx4, FDX1, LC3 and TH primary antibodies at 1:100 dilution overnight at 4 °C. Slices were then incubated with biotinylated anti-rabbit IgG secondary antibody at 1:500 dilution following by DAB staining for signal development. A light microscope was used to take images to do analysis in Fiji software (ImageJ2 software, version for Max OS X, https://imagej.net/software/fiji) through the IHC profiler. The IHC scores were set as high positive scores = 4.0, positive scores = 3.0, low positive scores = 2.0, negative scores = 1.0.

### Enzyme-linked Immunosorbent (ELISA) assay

Levels of IL-1ß, TNF-α, IL-10, MDA, GSH/GSSG, NADP^+^/NADPH in the supernatants of lysis of OB, Hippo, CS, COR, MB and CE of the brain tissues were measured using the ELISA commercial reagent kits according to the manufacturer’s instructions. All treatments were performed in triplicate in three independent experiments.

### Western blotting analysis

Expression levels of proteins in different brain tissues were assessed by western blotting analysis. The total protein was extracted using RIPA buffer supplemented with 1% protease inhibitor cocktail (Millipore, USA), and then determined the concentration by Bicinchoninic Acid (BCA) protein assay (Beyotime, Shanghai, China). The proteins were electrophoresized in a 10% sodium dodecyl sulfatepolyacrylamide gelelectrophoresis (SDS-PAGE) separation gel, transferred to PVDF membranes and then blocked in 5% non-fat milk at room temperature for 1 h. After that, membranes were washed with Tris buffered saline tween (TBST) and then incubated with primary antibodies, including caspase 3, caspase 8, caspase 1, GSDMD, MLKL, p-MLKL, RIP1, p-RIP1, GPX4, ACSL4, FDX1, HSP70, LC3 and beclin-1 at 4 ^o^C overnight. Membranes were washed with TBST and subsequently incubated with secondary antibodies at room temperature for 2 h. Finally, protein bends were exhibited by enhanced chemiluminenscence (ECL) solution (Millipore, USA). The same samples ran three times and the treatments were repeated independently at least three times.

### Behavioral tests

#### Open-field test

The open-field test was used to evaluate the locomotion and spontaneous activity of mice. Each mouse was gently placed in the center of a square field (40 × 40 cm) and moved freely for 5 min. A digital camera was used to record the movement of each mouse that would be analyzed with an animal motion tracking system (EthoVersion XT 17.0). The open-field test data were presented as the total moved distance and the time spent in center.

#### Suspension test

The suspension test was used to assess muscle strength of mice. The mouse was hoisted and timed with its two front paws from a horizontal wire (30 centimeters high from the ground and 0.1 centimeters in diameter) until its front paw released and fell off. Three tests were calculated for statistical analysis. The score was according to the hanging state of mice: both paws can hang = 3.0, one paw can hang = 2.0, neither paw can hang = 1.0. Each mouse was tested three times to get the average score.

#### Pole-climbing test

A self-made straight cylindrical rod with a length of 50 cm and an inner diameter around 1 cm was used to conduct the pole-climbing test. The rod is wrapped with gauze to prevent mice from slipping. Each mouse was gently put on the top of the pole with head up and then recorded via a digital camera. The time of the mouse turning towards the ground (time to orient down) and climbing down the pole to the ground (time to descend) were recorded. Each mouse was tested three times to get the average score.

#### Swim test

A container (20 × 20 × 30 cm) with water of 22–25 ^o^C and depth of 10 cm to do the swim test of mice. Each mouse was gently put into the container and recorded using a digital camera for 1 min. Each mouse was tested three times with an interval of 1 min to get the average score. The swim score was according to the study of GA Donnan [47]: continuous swimming movement = 3.0, occasional floating = 2.5, floating > 50% of time = 2.0, occasional swimming only = 1.5, occasional swimming using hind limbs while floating on side = 1.0, no use of limbs = 0.

### Data analysis

All data were displayed as the mean ± standard deviation (SD). Statistical analysis was performed using GraphPad Prism 6.0 Software. The two-tailed t tests were used to determine the statistical significance between the control and treatment group. Probability levels of < 0.05, < 0.01 and < 0.001were considered statistically significant.

### Electronic supplementary material

Below is the link to the electronic supplementary material.

Supplementary Material 1

## Data Availability

The datasets used and/or analyzed during the current study are available from the corresponding author on reasonable request.
