# Supplementary material for: The discovery of regional neurotoxicity-associated metabolic alterations induced by carbon quantum dots in brain of mice using a spatial metabolomics analysis
Source: Part Fibre Toxicol. 2024 Apr 10;21:19. doi: 10.1186/s12989-024-00580-y (PMC11005155; doi:10.1186/s12989-024-00580-y)
Supplement: Supplementary file 1 — Supplementary Material 1 [file 12989_2024_580_MOESM1_ESM.docx]

# Supporting Information

The discovery of regional neurotoxicity-associated metabolic alterations induced by carbon quantum dots in brain of mice using a spatial metabolomics analysis

Min Chen^1,#^, Siyuan Chen^1,#^, Xinyu Wang^1^, Zongjian Ye^1^, Kehan Liu^1^, Yijing Qian^1^, Meng Tang^1^ and Tianshu Wu^1,*^

^1^ Key Laboratory of Environmental Medicine and Engineering, Ministry of Education; School of Public Health, Southeast University, Nanjing 210009, P.R.China.

^2^ School of Medicine, Southeast University, Nanjing 210009, P.R.China.

^#^ Authors equally contributed to the work.

^*^ Author to whom correspondence should be addressed. E-Mail: [ninatswu@126.com](mailto:ninatswu@126.com) / ninatswu@seu.edu.cn

**Table S1: The summary of physicochemical characteristics of CQDs**

| QDs | Mean size by TEM (nm) | Mean size by DLS (nm) | ξ-potential (mV) | Excitation peak (nm) | Excitation peak (nm) |
| --- | --- | --- | --- | --- | --- |
| CQDs | 2.95 | ~84.2 | ~-25 | 380 | 480 |

**Table S2: Fatty acids identified in the AFADESI-MSI with brain distribution information in CQDs-treated mice and the control**

| Fatty acids | Formula | Measured *m*/*z* | Adduct | Ion mode | ppm | Regions of brain with significant difference |
| --- | --- | --- | --- | --- | --- | --- |
| FA(16:0) | C16H32O2 | 255.23259 | -H | negative | 1.441496 | OB, Hippo, CS, COR, MB, CE |
| FA(16:1) | C16H30O2 | 253.21660 | -H | negative | 2.792467 | Hippo, CS, CE |
| FA(18:0) | C18H36O2 | 283.26284 | -H | negative | 4.979163 | OB, Hippo, CS, COR, MB, CE |
| FA(18:1) | C18H34O2 | 281.24755 | -H | negative | 3.749085 | OB, Hippo, CS, COR, MB, CE |
| FA(20:1) | C20H38O2 | 309.27934 | -H | negative | 1.812607 | OB, Hippo, CS, COR, MB, CE |
| FA(20:2) | C20H36O2 | 307.26336 | -H | negative | 2.906529 |  |
| FA(20:3) | C20H34O2 | 305.24812 | -H | negative | 1.586276 | Hippo, CS, COR, MB, CE |
| FA(20:4) | C20H32O2 | 303.23247 | -H | negative | 1.605289 | OB, Hippo, CS, COR, MB, CE |
| FA(20:5) | C20H30O2 | 301.21676 | -H | negative | 1.793615 | OB, CE |
| FA(22:1) | C22H42O2 | 337.31039 | -H | negative | 2.405452 |  |
| FA(22:3) | C22H38O2 | 333.27910 | -H | negative | 2.424803 |  |
| FA(22:4) | C22H36O2 | 331.26360 | -H | negative | 1.967649 | Hippo, CS, COR, MB, CE |
| FA(22:5) | C22H34O2 | 329.24768 | -H | negative | 2.792222 | OB, Hippo, CS, CE |
| FA(22:6) | C22H32O2 | 327.23228 | -H | negative | 2.073598 | Hippo, CS, COR, MB, CE |
| FA(24:1) | C24H46O2 | 365.34115 | -H | negative | 3.702844 |  |

Olfactory bulb (OB), Cortex (COR), Hippocampus (Hippo), Corpus Striatum (CS), Midbrain (MB), Cerebellum (CE), Nucleus Accumbens (NAcc), Habenula Nucleus (HB) and Zona Incerta (ZI). The instrument model: AFADESI-MSI platform (Beijing Victor Technology Co., LTD, Beijing, China) in tandem with a Q-Orbitrap mass spectrometer (Q Exactive, Thermo Scientific, U.S.A.); Mass resolution: 60000.

**Table S3: Identified potential neurotoxicity-associated metabolic enzymes affected by CQDs in brain of mice**

| Enzyme | EC number | Metabolic pathway | Related metabolites | Function |
| --- | --- | --- | --- | --- |
| SMS | EC:2.5.1.22 | Arginine and proline metabolism, ß-Alanine metabolism | Spermidine, Spermine | Catalyzes the production of spermine from spermidine and decarboxylated S-adenosylmethionine (dcSAM). |
| ACOT2 | EC:3.1.2.2 | Biosynthesis of unsaturated fatty acids, Fatty acid elongation | FA(22:6), FA(20:3), FA(20:4), FA(20:5), FA(18:1) | catalyzes the hydrolysis of acyl-CoAs to the free fatty acid and coenzyme A (CoASH), providing the potential to regulate intracellular levels of acyl-CoAs, free fatty acids and CoASH. |
| BAAT | EC:2.3.1.65 | Biosynthesis of unsaturated fatty acids, Primary bile acid biosynthesis | FA(16:0), FA(18:0) | Catalyzes the amidation of bile acids (BAs) with the amino acid taurine. |
| GLS | EC:3.5.1.2 | Glutamine and glutamate metabolism, Glutamatergic synapse | Glutamate, Glutamine | Catalyzes the first reaction in the primary pathway for the renal catabolism of glutamine; Plays a role in maintaining acid-base homeostasis; Regulates the levels of the neurotransmitter glutamate, the main excitatory neurotransmitter in the brain. |
| GS | EC:6.3.1.2 | Glutamine and glutamate metabolism, Glutamatergic synapse | Glutamine, Glutamate | catalyzes the ATP-dependent conversion of glutamate and ammonia to glutamine. |

**Table S4: The details of antibodies**

| Abbreviation | Description | Manufactures | Catalog NO. |
| --- | --- | --- | --- |
| Casp3 | Caspase 3 | ZENBIO | 341034 |
| Casp8 | Caspase 8 | ZENBIO | R23731 |
| Casp1 | Caspase 1 | ThermoScientific | PA5-87536 |
| p-MLKL | Phospho- mixed lineage kinase domain-like-Ser454 | ABclonal | AP1173 |
| t-MLKL | mixed lineage kinase domain-like | ABclonal | A21894 |
| p-RIP1 | phospho-receptor interacting protein-1 | ABclonal | AP1314 |
| t-RIP1 | receptor interacting protein-1 | ABclonal | A7414 |
| GPX4 | glutathione peroxidase 4 | ABclonal | A1933 |
| FDX1/ADX | ferredoxin 1/ Adrenodoxin | ABclonal | A20895 |
| HSP70 | heat shock protein 70 | ZENBIO | 382481 |
| Beclin-1 | Beclin-1 | Cell Signaling Technology | D40C5 |
| GSDMD | gasdermin D | ABclonal | A20728 |
| ACSL4 | acyl coenzyme A synthetase long chain family member 4 | ABclonal | A16848 |
| LC3 | microtubule associated protein 1A/1B light chain 3 | ZENBIO | 381544 |
| ACOT2 | acyl-CoA thioesterase 2 | ABclonal | A14390 |
| BAAT | bile acid-Coenzyme A: amino acid N-acyltransferase | ABclonal | A7646 |
| SMS | Spermine Synthase | ZENBIO | 824889 |
| GLUL/GS | Glutamine Synthetase (GLUL) | ABclonal | A5437 |
| GAPDH | glyceraldehyde-3-phosphate dehydrogenase | ABclonal | A19056 |
| TH | Tyrosine Hydroxylase | ABclonal | A5079 |

**Table S5: Abbreviations**

| Abbreviation | Description |
| --- | --- |
| ACOT2 | acyl-CoA thioesterase 2 |
| ACSL4 | acyl coenzyme A synthetase long chain family member 4 |
| AD | Alzheimer's disease |
| AFADESI-MSI | airflow-assisted desorption electrospray ionization mass spectrometry imagin |
| ALS | amyotrophic lateral sclerosis |
| BAAT | bile acid-Coenzyme A: amino acid N-acyltransferase |
| BCA | Bicinchoninic Acid Assay |
| CE | cerebellum |
| CNS | central nervous system |
| COR | cortex |
| CQDs | carbon quantum dots |
| CS | corpus striatum |
| DAB | 3,3'-Diaminobenzidine |
| DHA | docosahexaenoic acid |
| ECL | enhanced chemiluminenscence |
| ELISA | Enzyme-linked Immunosorbent Assay |
| FDX1 | ferredoxin 1 |
| FT-IR | fourier transform infrared |
| GAPDH | glyceraldehyde-3-phosphate dehydrogenase |
| GLS | glutaminase |
| GPX4 | glutathione peroxidase 4 |
| GS | glutamine synthetase |
| GSDMD | gasdermin D |
| GSDME | gasdermin E |
| GSH/GSSG | Reduced glutathione/Oxidized glutathione |
| H&E | hematoxylin and eosin |
| HD | Huntington's disease |
| HIP | hippocampus |
| HR-TEM | high-resolution transmission electron microscope |
| HSP70 | heat shock protein 70 |
| IHC | Immunohistochemistry |
| IL-10 | Interleukin-10 |
| IL-1ß | Interleukin-1β |
| KEGG | kyoto encyclopediaof genesand genomes |
| LC3 | microtubule associated protein 1A/1B light chain 3 |
| LHB | Lateral Habenula Nucleus |
| MB | midbrain |
| MDA | malondialdehyde |
| MLKL | mixed lineage kinase domain-like |
| MS | mass spectrometry |
| MSI | mass spectrometry imaging |
| NAcc | Nucleus Accumbens |
| NADP+/NADPH | nicotinamide adenine dinucleotidephosphate |
| OB | Olfactory bulb |
| PCDs | programmed cell deaths |
| PD | Parkinson disease |
| PL | photoluminescent |
| PLS-DA | partial least squares discriminant analysis |
| p-MLKL | phospho-mixed lineage kinase domain-like |
| p-RIP1 | phospho-receptor interacting protein-1 |
| RIP1 | receptor interacting protein-1 |
| SD | standard deviation |
| SDS-PAGE | sulfatepolyacrylamide gelelectrophoresis |
| SMS | spermine synthase |
| SNpc | substantia nigra pars compacta |
| TBST | Tris buffered saline tween |
| TCA | Trichloroacetic acid |
| TH | tyrosine hydroxylase |
| TNF-α | tumor necrosis factor-α |
| TUNEL | terminal-deoxynucleotidyl transferase mediated nick end labelling |
| ZI | Zona Incerta |


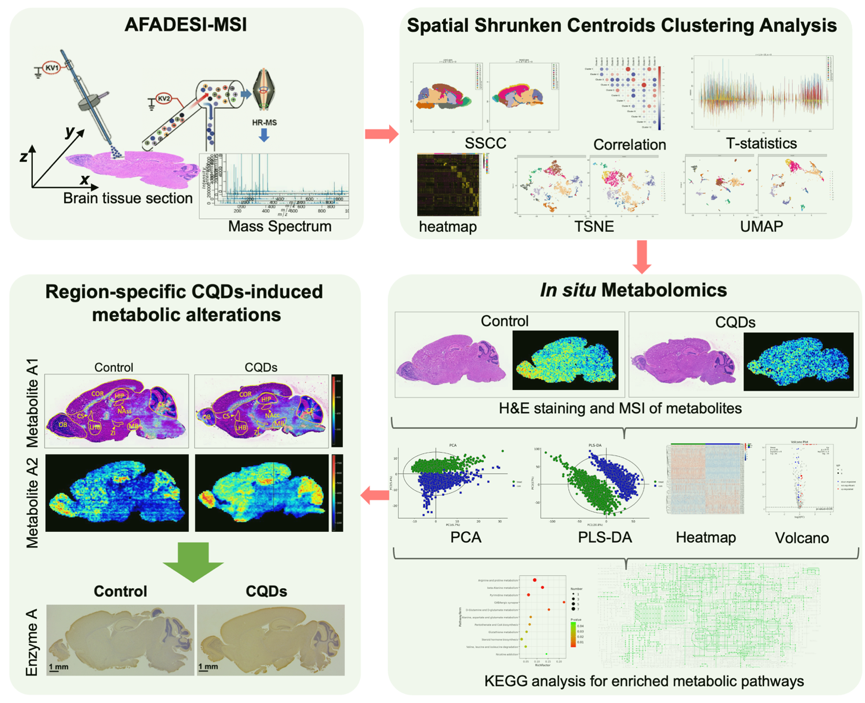


**Figure S1: The strategy on the discovery of metabolic alterations caused by intranasally exposed CQDs in brain of mice.**

**
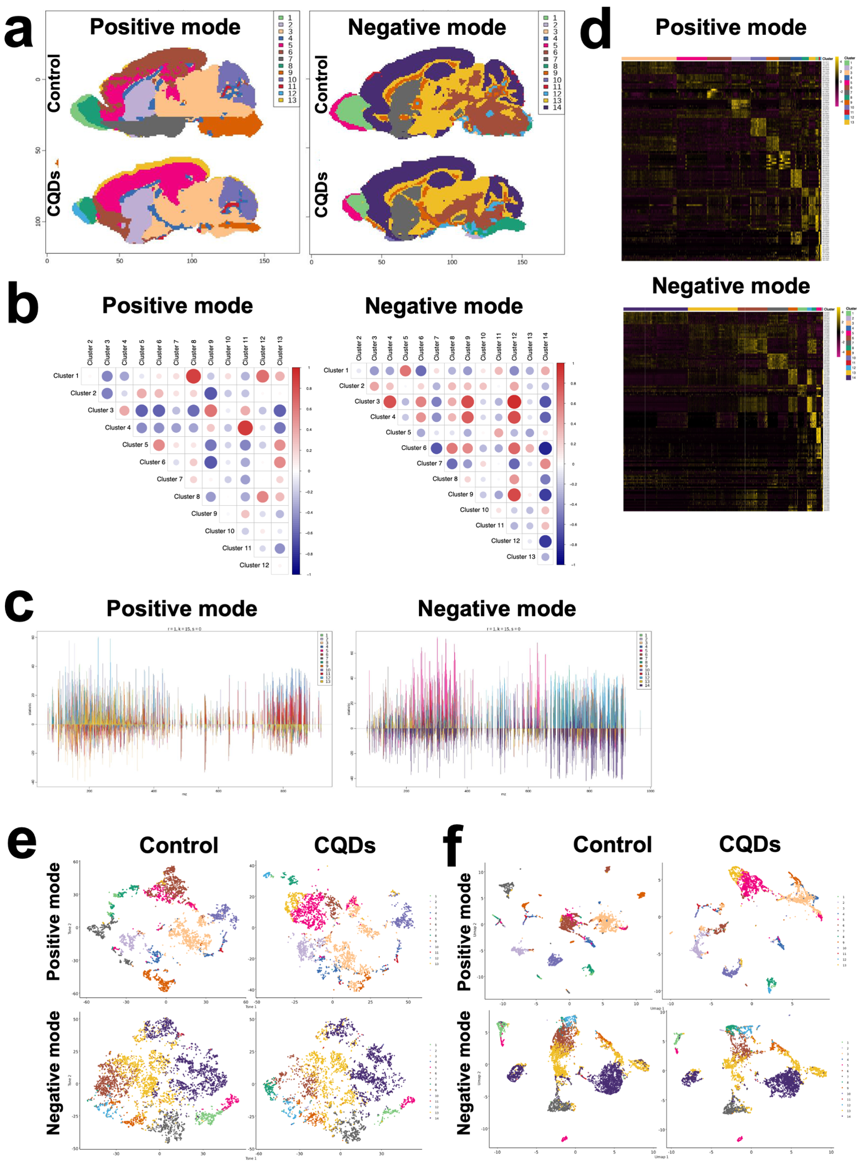
**

**
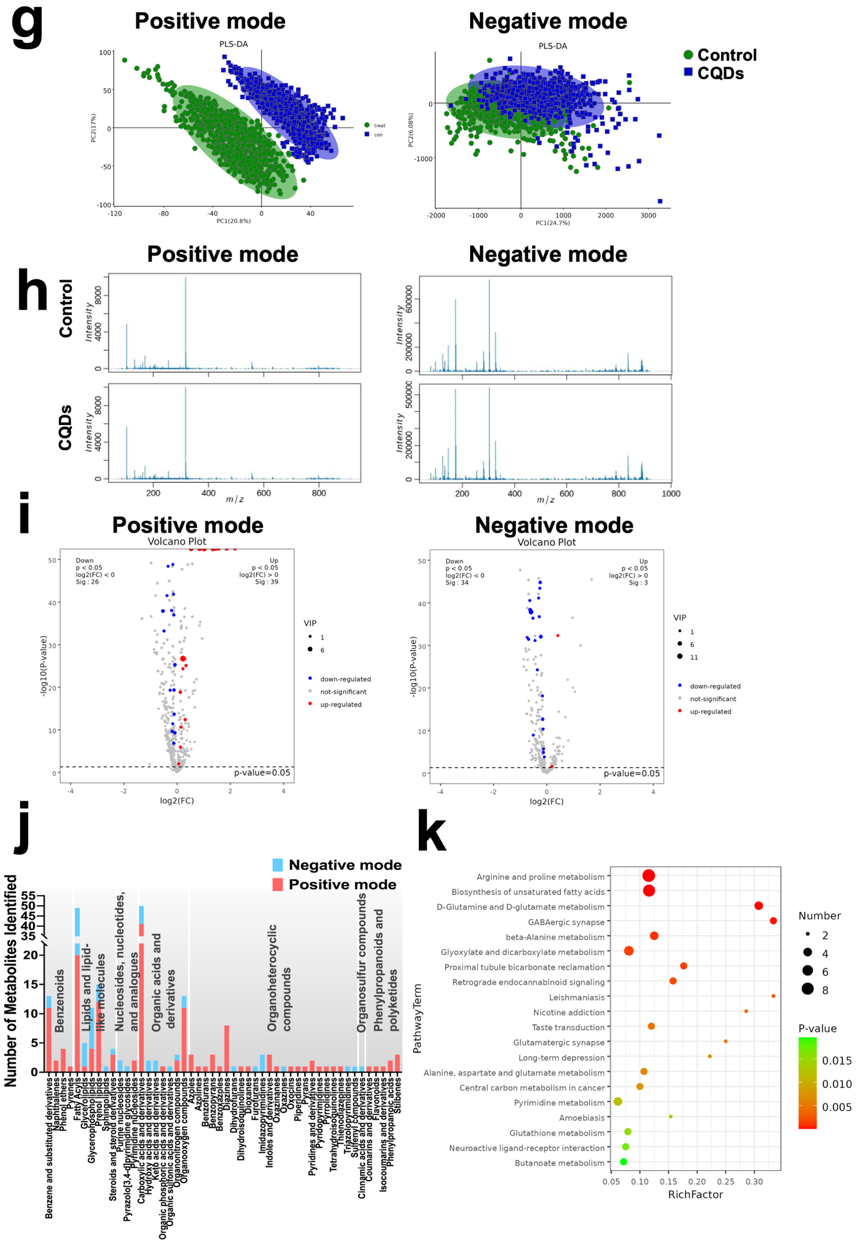
**

**Figure S2: All metabolites detected by AFADESI-MSI method on brain tissue sections of mice and metabolites significantly changed by CQDs exposure.** (a) Spatial shrunken centroids clustering (SSCC) analysis; Correlation (b), T statistics (c), Heatmap (d), T-Distributed Stochastic Neighbor Embedding (TSNE) analysis (e), Uniform Manifold Approximation and Projection for Dimension Reduction (UMAP) analysis (f) of clusters based on metabolites in the brain tissues identified from positive and negative modes; PLS-DA models (g) and representative mass spectra (h) of the brain tissues based on positive and negative ion modes; Volcano maps (i) and Quantitative analysis based on different super classes and classes (j) of metabolites significantly changed by CQDs; (k) Bubble chart for the enrichment analysis of TOP20 metabolic pathways based on all metabolites significantly changed by CQDs.


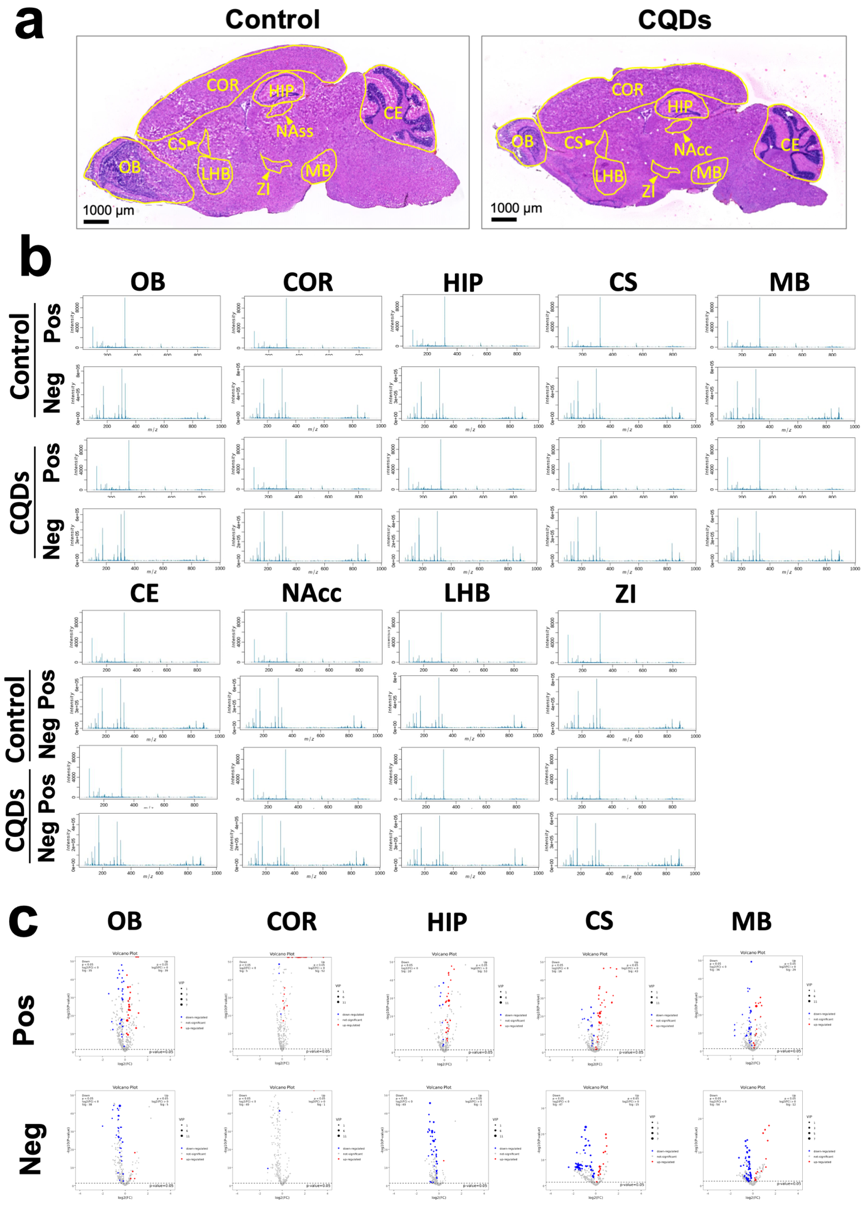


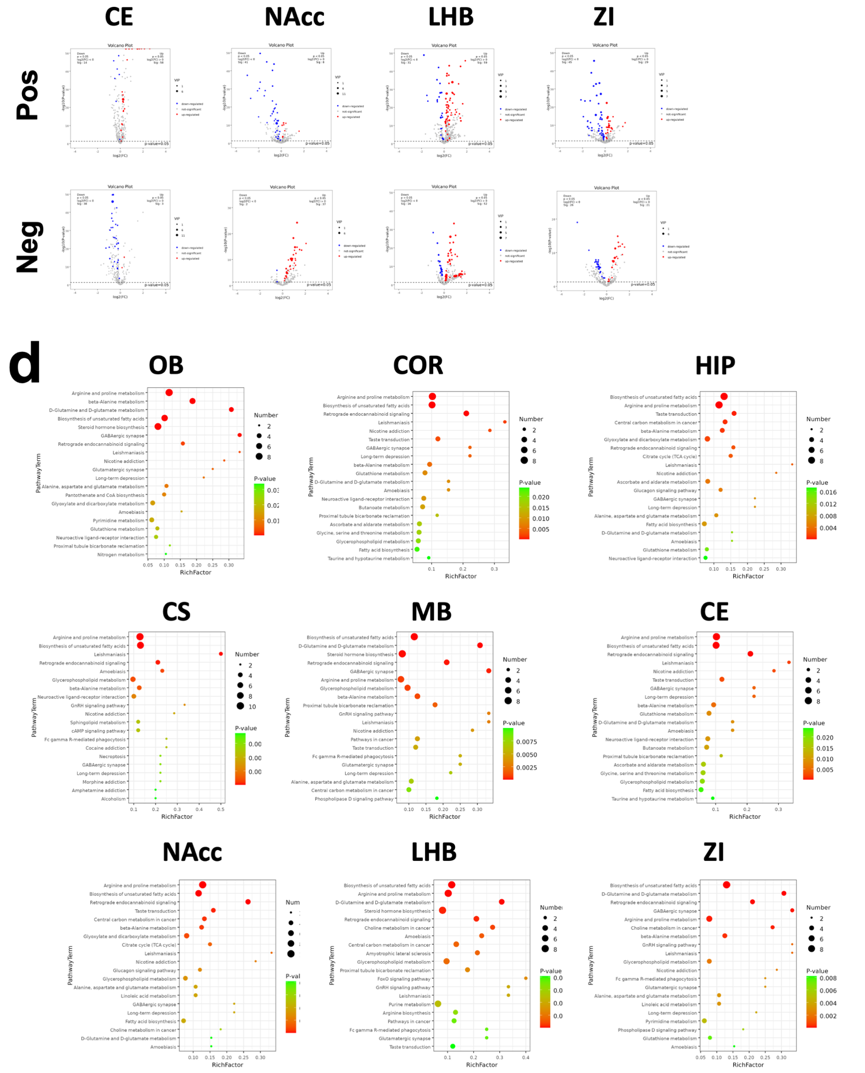


**Figure S3: Metabolites detected by AFADESI-MSI method significantly changed by CQDs exposure in different regions of the brain of mice.** (a) H&E images and the selected regions of the brain tissue; (b) Representative mass spectra of different regions of the brain tissue in positive and negative ion modes; (c) Volcano maps of metabolites significantly changed by CQDs identified from positive and negative ion modes; (d) Bubble charts for the enrichment analysis of TOP20 metabolic pathway based on all metabolites significantly changed by CQDs in different regions of the brain. Olfactory bulb (OB), Cortex (COR), Hippocampus (HIP), Corpus Striatum (CS), Midbrain (MB), Cerebellum (CE), Nucleus Accumbens (NAcc), Lateral Habenula Nucleus (LHB) and Zona Incerta (ZI). Data are showed as mean+SD of three independent experiments. (**P*<0.05, ***P*<0.01, ****P*<0.001).

**
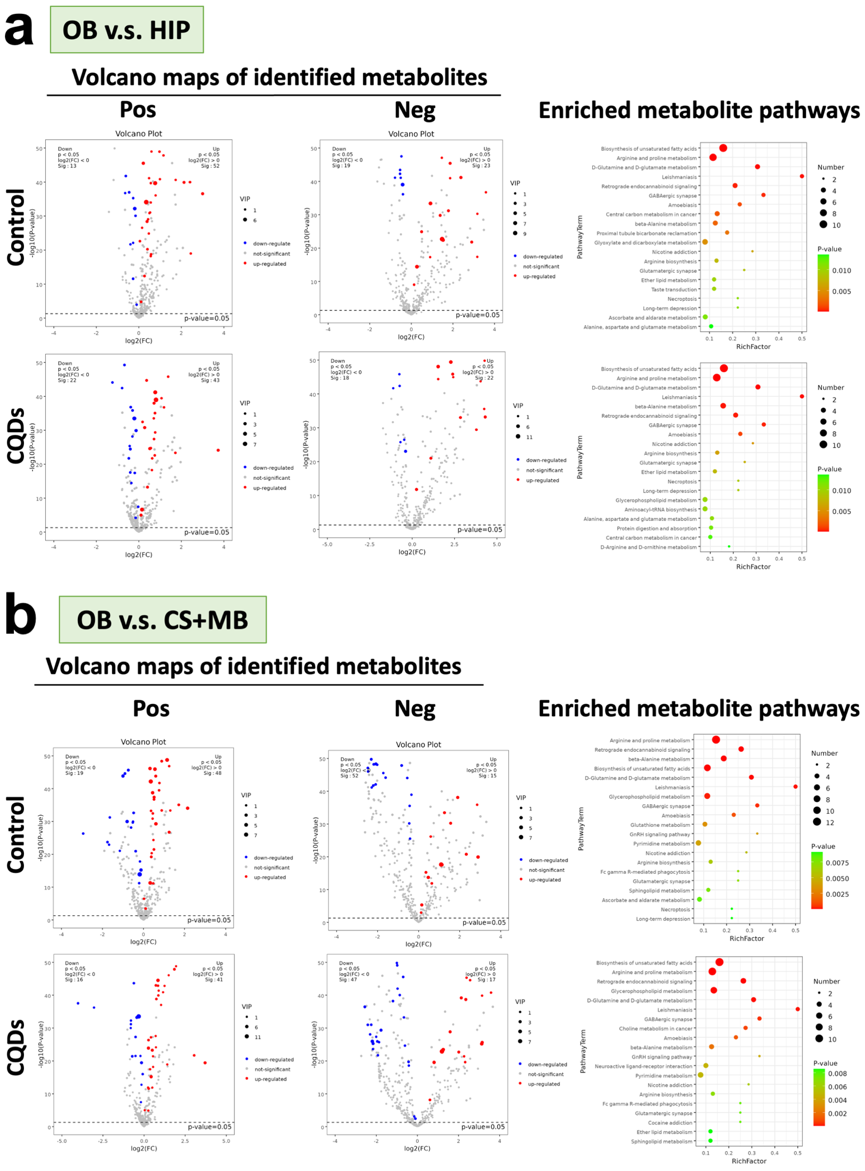
**

**
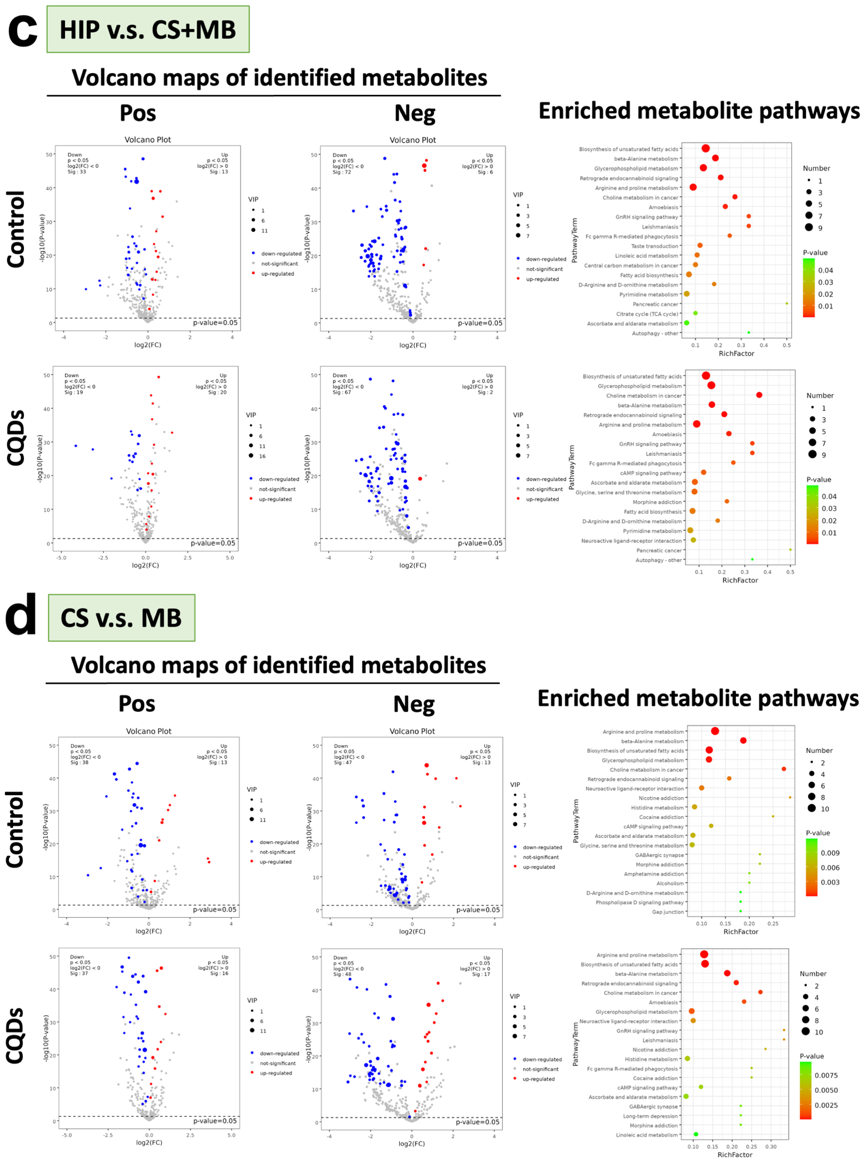
**

**Figure S4: The spatial distribution of metabolites within the brain of mice treated without/with CQDs.** Volcano maps of metabolites identified from positive and negative ion modes, and corresponding bubble charts for the enrichment analysis of TOP20 metabolic pathway between OB and HIP (a), OB and CS+MB (b), HIP and CS+MB (c), and CS and MB (d) of brain of mice treated without/with CQDs. Olfactory bulb (OB), Hippocampus (HIP), Corpus Striatum (CS) and Midbrain (MB).


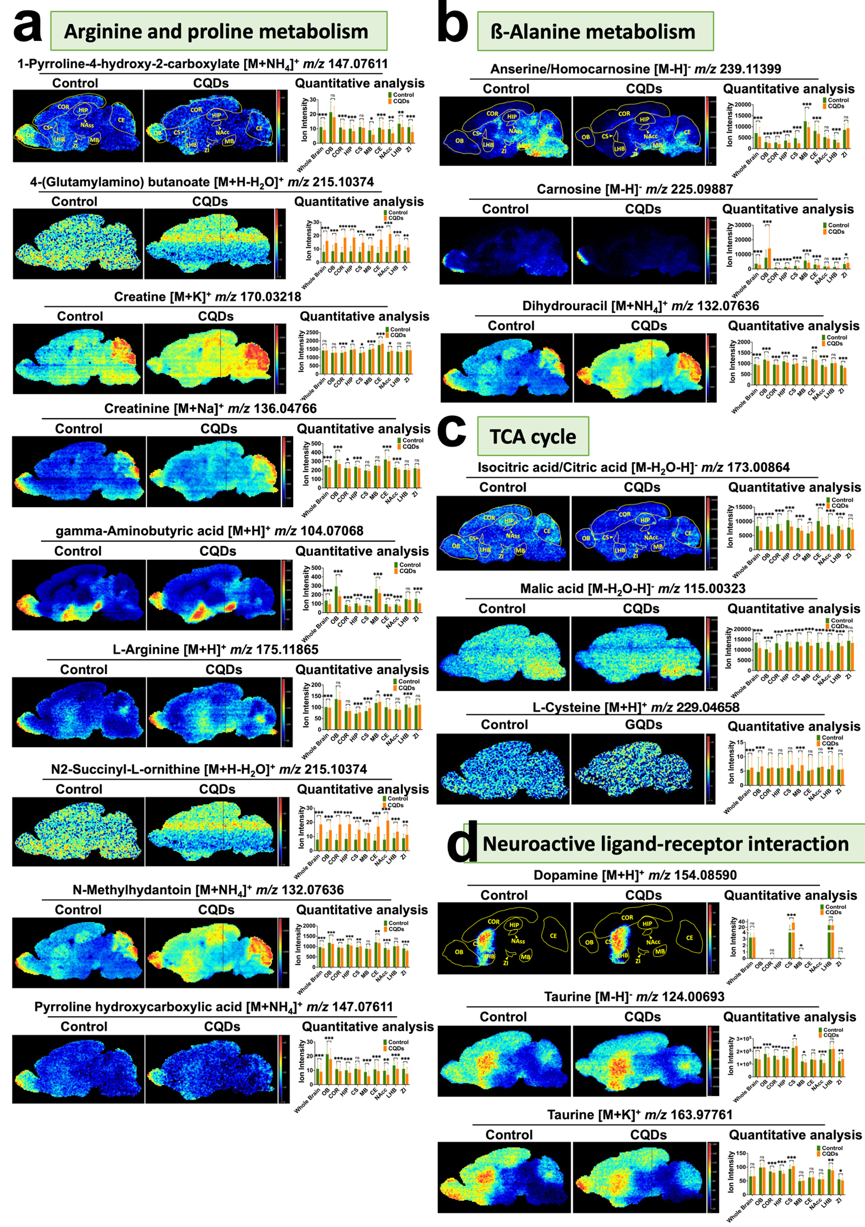


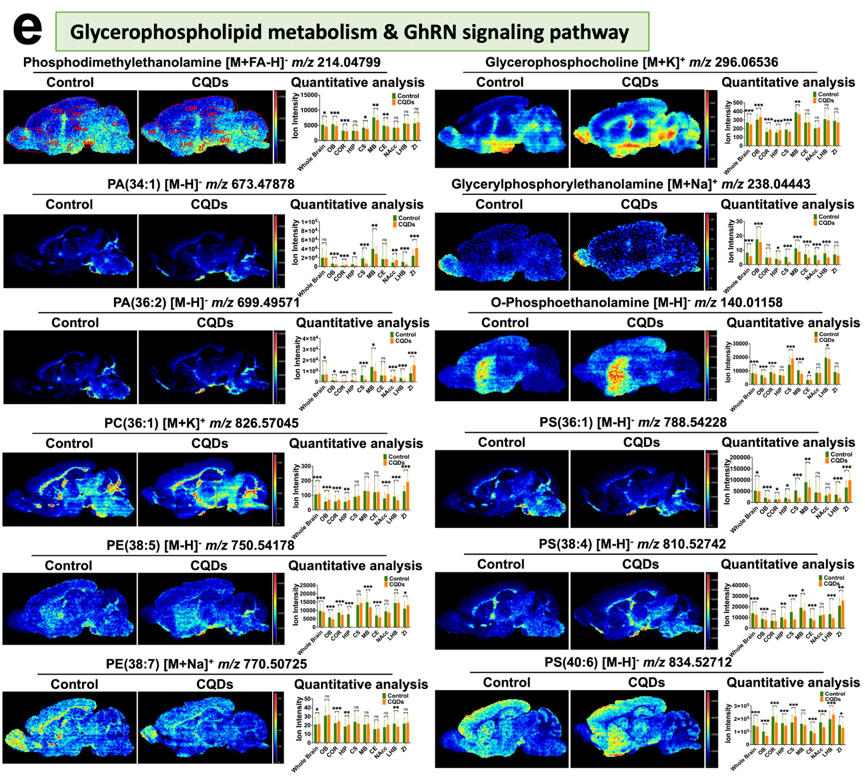


**Figure S5: In situ visualization of critical metabolites in corresponding enriched metabolic pathways significantly changed by CQDs in different regions of the brain of mice based on AFADESI-MSI data.** MS images and levels of metabolites in arginine and proline metabolism (a), ß-Alanine metabolism (b), TCA cycle (c), neuroactive ligand-receptor interaction (d), and glycerophospholipid metabolism and GhRN signaling pathway (e) in different regions of brain. Olfactory bulb (OB), Cortex (COR), Hippocampus (HIP), Corpus Striatum (CS), Midbrain (MB), Cerebellum (CE), Nucleus Accumbens (NAcc), Lateral Habenula Nucleus (LHB) and Zona Incerta (ZI). Data are showed as mean+SD of three independent experiments. (**P*<0.05, ***P*<0.01, ****P*<0.001).


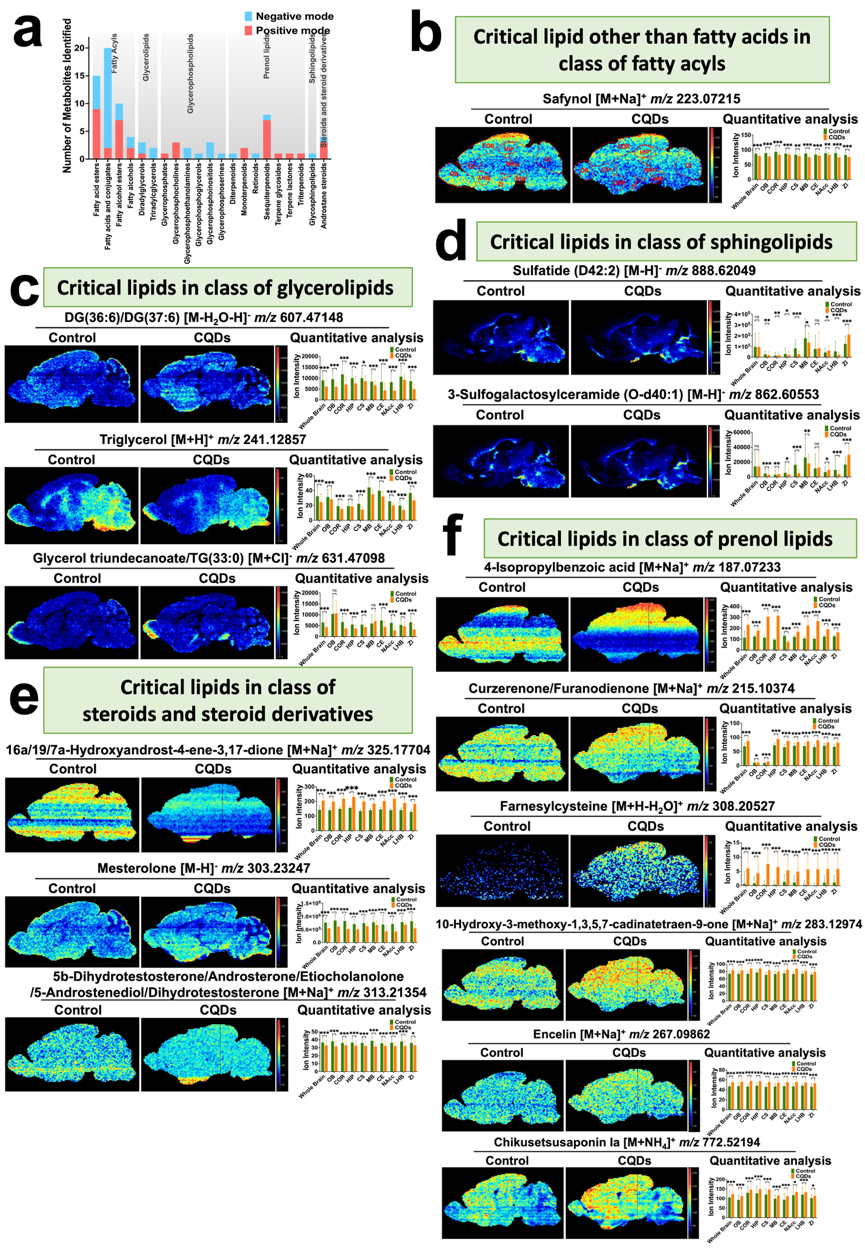


**Figure S6: In situ visualization of critical lipids and lipid-like molecules significantly changed by CQDs based on AFADESI-MSI data.** (a) Quantitative analysis of lipids and lipid-like molecules significantly changed by CQDs identified from positive and negative ion models being divided into different classes and subclasses; MS images and levels of critical lipids other than fatty acids in class of fatty acyls (b), glycerolipids (c), sphingolipids (d), steroids and steroid derivatives (e), and prenol lipids (f) in different regions of brain. Olfactory bulb (OB), Cortex (COR), Hippocampus (HIP), Corpus Striatum (CS), Midbrain (MB), Cerebellum (CE), Nucleus Accumbens (NAcc), Lateral Habenula Nucleus (LHB) and Zona Incerta (ZI). Data are showed as mean+SD of three independent experiments. (**P*<0.05, ***P*<0.01, ****P*<0.001).


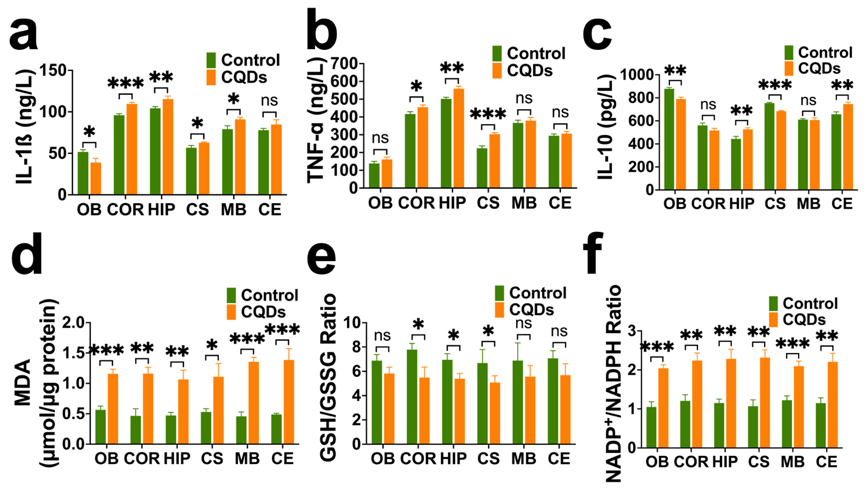


**Figure S7: CQDs induced neuroinflammation and redox interruption in brain of mice.** (a, b, c) The levels of IL-1ß, TNF-α and IL-10, MDA content (d), GSH/GSSG ratio (e) and NADP^+^/NADPH ratio (f) in different regions of the brain of mice treated with CQDs. Olfactory bulb (OB), Cortex (COR), Hippocampus (HIP), Corpus Striatum (CS), Midbrain (MB), Cerebellum (CE). Data are showed as mean+SD of three independent experiments. (**P*<0.05, ***P*<0.01, ****P*<0.001).


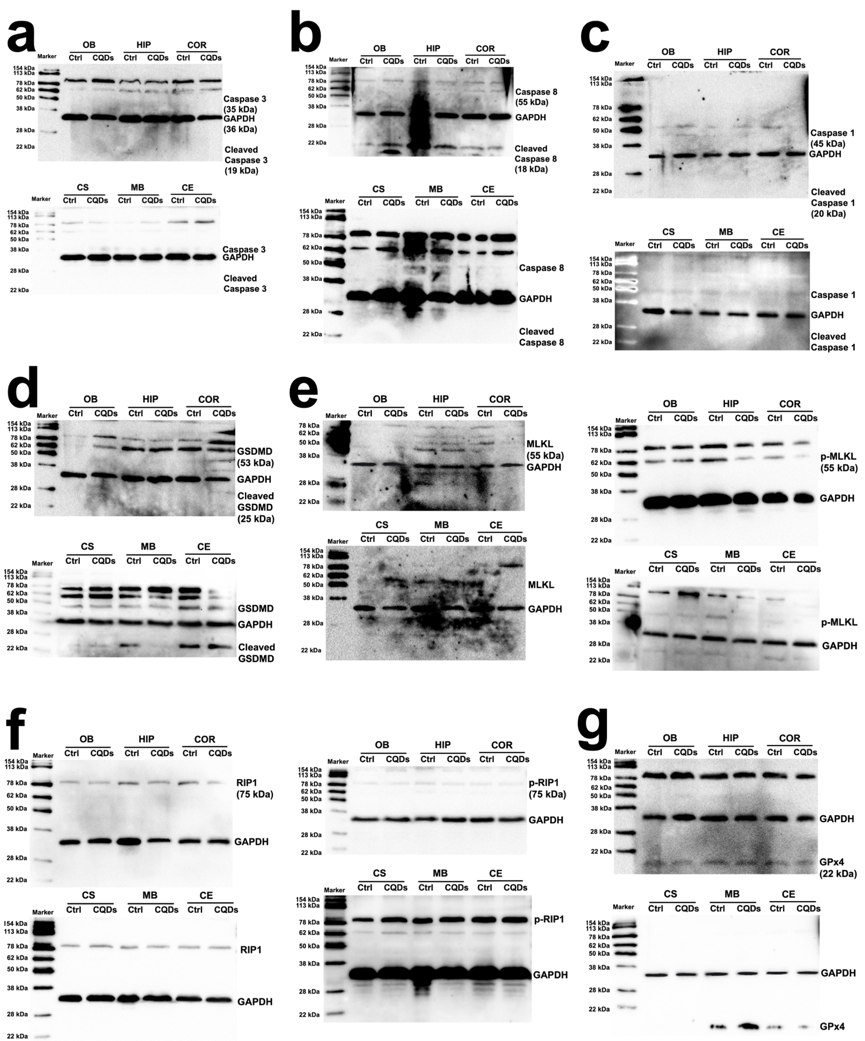


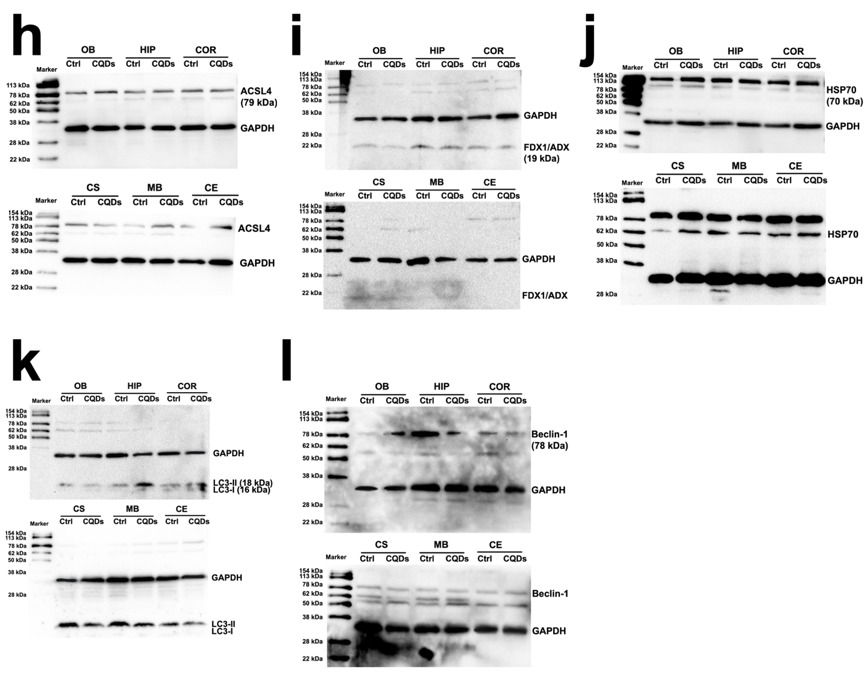


**Figure S8: CQDs altered the expressions of protein biomarkers of multiple programmed cell deaths in brain of mice.** Protein expressions of caspase 3 (a), caspase 8 (b), caspase 1 (c), GSDMD (d), t/p-MLKL(e), t/p-RIP1 (f), GPx4 (g), ACSL4 (h), FDX1 (i), HSP70 (j), LC3-I/II (k) and Becline-1 (l) in different regions of brain of mice treated with CQDs. Olfactory bulb (OB), Cortex (COR), Hippocampus (HIP), Corpus Striatum (CS), Midbrain (MB) and Cerebellum (CE).

**The methods of AFADESI-MSI**

**Experimental reagents**

MS-grade acetonitrile (Thermo Fisher, USA); Purified water (Watsons, Hongkong, China); Formic acid (Merck, Germany); Tissue freezing medium (Leica Microsystem, Germany); Eosin Y-solution 0.5% aqueous (St. Louis, MO, USA); Hematoxylin (St. Louis, MO, USA); embedding glue (Cryo-Gel frozen section embedding glue, Leica Microsystem, Germany, 14020108926).

**Experimental Instruments**

Cryostat microtome (Leica CM 1950, Leica Microsystem, Germany); positive charge desorption plate (Thermo Scientific, USA); AFADESI-MSI platform (Beijing Victor Technology Co., LTD, Beijing, China); Q-Orbitrap mass spectrometer (Q Exactive, Thermo Scientific, USA).

**Sample preparation**

**Embedded:** Prepare a plastic container slightly larger than the mouse brain and centrifuge the embedding glue to remove air bubbles from the embedding glue. Flatten the bottom of the plastic container with the embedding glue, place the dissected intact mouse brain on top of the flattened embedding glue, and continue to slowly add the embedding glue until it encapsulates the entire brain. The embedded samples were stored at -80 °C before being sectioned.

**Frozen section:** The samples were cut into consecutive sagittal slices of 10 μm thickness using a cryostat microtome. The slices were then thaw-mounted on a positive charge desorption plate and desiccated at -20 °C for 1 hour, followed by 0.5 hours at room temperature before mass spectrometry imaging (MSI) analysis. A slice adjacent to it was reserved for hematoxylin-eosin (H&E) staining.

**MSI analysis**

The solvent formula used was ACN/H2O (8:2) in negative mode and ACN/H2O (8:2, 0.1% FA) in positive mode. The solvent flow rate was 5 μL/min, the transporting gas flow rate was 45 L/min, the spray voltage was set at 7 kV, and the distance between the sample surface and the sprayer was 3 mm, as was the distance from the sprayer to the ion transporting tube. The mass spectrometer resolution was set to 60,000 with a mass range of 70-1000 Da. The automated gain control target was set to 2E6, and the maximum injection time was 200 ms. The S-lens voltage was set to 55 V, and the capillary temperature was maintained at 350 °C. The MSI experiment was conducted by continuously scanning the surface of the sample section in the x direction at a constant rate of 0.2 mm/s, with a 100 μm vertical step in the y direction. The raw files have been collected.

**Data Processing**

The .raw files were converted to .imzML format using imzMLConverter 2. The resulting files were then imported into MSiReader, an open-source interface for viewing and analyzing high-resolution MS imaging files on the Matlab platform. Background subtraction was performed using the Cardinal 3 software package. Ion image reconstructions were carried out using MSiReader. Total ion count normalization (TIC) was used to normalize all MS images in each pixel. High-spatial resolution H&E images were used to precisely extract region-specific MS profiles. A supervised statistical analytical method, orthogonal partial least squares discrimination analysis (OPLS-DA), was employed to screen the discriminating endogenous molecules of different tissue microregions. The Variable Importance of Projection (VIP) values obtained from the OPLS-DA model were used to rank the overall contribution of each variable to group discrimination. The VIP value indicates the degree of importance in classifying sample categories based on the first two principal components of the OPLS-DA model. A variable has a significant effect if its VIP is greater than 1. To verify the significance of the difference between groups, a two-tailed Student's T-test was conducted. Differential metabolites were selected based on VIP values greater than 1.0 and p-values less than 0.05.

In addition, we performed T-distributed stochastic neighbor embedding (t-SNE) and uniform manifold approximation and projection for dimensionality reduction on the MS data in each pixel. We also applied Spatial Shrunken Centroids Clustering (SSCC) to cluster the MSI data and separate the samples based on differences in ion abundance in each pixel.

**Analyte Identification**

The ions detected by AFADESI were annotated using the pySM 5 pipeline and an in-house SmetDB database (Lumingbio, Shanghai, China).
